# Tool evaluation for the detection of variably sized indels from next generation whole genome and targeted sequencing data

**DOI:** 10.1371/journal.pcbi.1009269

**Published:** 2022-02-17

**Authors:** Ning Wang, Vladislav Lysenkov, Katri Orte, Veli Kairisto, Juhani Aakko, Sofia Khan, Laura L. Elo

**Affiliations:** 1 Turku Bioscience Centre, University of Turku and Åbo Akademi University, Turku, Finland; 2 Department of Pathology, Laboratory Division, Turku University Hospital, Turku, Finland; 3 Department of Genomics, Laboratory Division, Turku University Hospital, Turku, Finland; 4 Institute of Biomedicine, University of Turku, Finland; Temple University, UNITED STATES

## Abstract

Insertions and deletions (indels) in human genomes are associated with a wide range of phenotypes, including various clinical disorders. High-throughput, next generation sequencing (NGS) technologies enable the detection of short genetic variants, such as single nucleotide variants (SNVs) and indels. However, the variant calling accuracy for indels remains considerably lower than for SNVs. Here we present a comparative study of the performance of variant calling tools for indel calling, evaluated with a wide repertoire of NGS datasets. While there is no single optimal tool to suit all circumstances, our results demonstrate that the choice of variant calling tool greatly impacts the precision and recall of indel calling. Furthermore, to reliably detect indels, it is essential to choose NGS technologies that offer a long read length and high coverage coupled with specific variant calling tools.

## Author summary

The development of next generation sequencing (NGS) technologies and computational algorithms enabled the large scale, simultaneous detection of a wide range of genetic variants, such as single nucleotide variants as well as insertions and deletions (indels), which may confer potential clinical significance. Recently, many studies have been conducted to evaluate variant calling tools for indel calling. However, the optimal indel size range for different variant calling tools remains unclear. A good benchmarking dataset for indel calling evaluation should contain biologically representative high-confident indels with a wide size range and preferably come from various sequencing settings. In this article, we created a semi-simulated whole genome sequencing dataset where the sequencing data were computationally generated. The indels in the semi-simulated genome were incorporated from a real human sample to represent biologically realistic indels and to avoid the inclusion of variants due to potential technical sequencing errors. Furthermore, we used three real-world NGS datasets generated by whole genome or targeted sequencing to further evaluate our candidate tools. Our results demonstrated that variant calling tools vary greatly in calling different sizes of indels. Deletion calling and insertion calling also showed differences among the tools. The sequencing settings in coverage and read length also had a great impact on indel calling. Our results suggest that the accuracy of indel calling was dependent on the combination of a variant calling tool, indel size range, and sequencing settings.

## Introduction

Next-generation sequencing (NGS) has developed rapidly in recent decades. Compared with traditional Sanger capillary electrophoresis sequencing or other directed polymerase chain reaction-based screening methods, it provides a more efficient and affordable way to detect genomic variants in large scale [1–3]⁠. NGS is widely used in research and in the clinic [4–7] and consists of techniques to cover the whole genome, i.e., whole genome sequencing (WGS), exome regions only, i.e., whole exome sequencing (WES), or certain genomic regions, i.e., targeted gene panel sequencing.

High-throughput NGS enables researchers to simultaneously identify large numbers of single nucleotide variants (SNVs) and insertions and deletions (indels), as well as other types of genomic aberrations, such as inversions and translocations. Indels are the second most common variant type in the human genome after SNVs. They are also the most common type of structural variants (SVs), defined as genomic variants > 50bp [8,9]⁠. Indels have been implicated in many diseases, such as Parkinson’s disease and cancers, thus their detection in the human genome is significant for clinical research [4,10]⁠.

To date, a number of computational tools for variant calling from NGS data have been published. They utilize different information such as concise idiosyncratic gapped alignment report (CIGAR) strings from binary alignment map (BAM) files and build their underlying algorithms based on paired-end sequencing reads, split-read, *de novo* sequence assembly, gapped sequence alignment, or machine learning to detect indels. Paired-end reads methods, based on paired-end sequencing, use discordantly mapped paired-end reads to identify indel breakpoints, which are the junctions that define structurally variable genomic segments [11]. In essence, paired-end reads that are mapped further, or closer than the expected insert size in the library, may indicate that an indel occurred between the alignments of the two paired-end reads [11]⁠. However, repeat regions in genomes, or SNVs near indel breakpoints, may influence the accuracy of indel calling results [11,12]. Methods based on split-read take reads that span the breakpoint of an indel as evidence to identify the indel at a single nucleotide level [13]. However, the short read length produced by NGS leads to even shorter split reads, which limit the alignment and make it hard to obtain a sufficient read depth around the indel breakpoint to confidently call an indel [12]⁠. Sequence assembly methods include *de novo* sequence assembly and local re-assembly. *De novo* sequence assembly assembles short reads into longer contigs, enabling a fine-scale discovery of large indels, especially novel sequence insertions [14,15]. Local re-assembly takes reads around a potential variant site with a reference sequence to re-build a haplotype and then generates an accurate variant allele and the corresponding genotype [16,17]. However, *de novo* sequence assembly methods require more computational resources and are prone to assembly errors [12]. Gapped sequence alignment-based methods use the alignment results from a gapped aligner, such as the Burrows-Wheeler Aligner (BWA) [18]⁠, and apply probabilistic models to make indel calls [16,19]. The mapping conditions of each base of reads from the input file provide evidence of indels. These methods require that the indels are contained within a read with accurate mapping conditions from the read alignment step [20]. These methods are sufficient for detecting small indels that are fully covered by a single read length but unsatisfactory for identifying indels longer than the read length [20]. Currently, as each of these methods has its own limitations, many variant calling tools use a combination of methods to detect a wider spectrum of indels [17,21]. Lately, machine learning methods such as the random forest model and the deep convolutional neural network have been applied in some of the variant calling tools to detect indels [22,23]⁠.

In recent years, many studies have been conducted to evaluate variant calling tools for indel calling, but these studies have performed evaluations with only a limited selection of sequencing data types or have only covered a limited indel size range. Sandmann et al. evaluated eight variant calling tools with both real and simulated non-matched targeted NGS data by evaluating the sensitivities, positive predictive values, F1 scores, and other metrics of tools for calling SNVs and indels smaller than 50bp [24]. Supernat et al. compared three variant calling tools with the WGS data of the well-known human individual NA12878 regarding variant calling precision, recall, and F1 score in calling SNVs and short indels [25]. Zhao et al. evaluated three variant calling tools with real and simulated human germline WGS data by comparing the precisions, recalls, and F1 scores of candidate tools with different genome contexts [26]. However, these evaluation studies focused only on variant calling tools for calling small-size indels with a certain type of NGS data, and the performance of tools for calling larger-size indels remains unknown. For large indel calling evaluation, Pei et al. used 14 next-generation and third-generation sequencing datasets to evaluate the precision rates, recall rates, and computation costs of 11 variant calling tools with both germline and somatic variant calling, but the effect of the varying indel size was not evaluated [27]. Kosugi et al. comprehensively evaluated 69 SV detection algorithms for different types of SVs by testing the representative tools with five simulated WGS datasets and a real WGS dataset for human individual NA12878 by using multiple evaluation metrics [28]. Cameron et al. selected 10 SV calling tools to evaluate their precision-recall rates, running times, concordances, and quality scores by using three real WGS datasets and *in silico* datasets with different sequencing settings [29]. In those studies, comprehensive evaluations of variant calling tools were performed but focused exclusively on SVs. The best performance of variant calling tools for a certain size range of indels, such as indels around 50bp, still remains unclear. Each algorithm uses different evidence from sequencing data to detect the breakpoint of an indel. Therefore, the best-detectable indel size range of algorithms might vary. It is important to determine the best-detectable indel size range of different tools with different sequencing methods: a wrong selection may result in missed or false positive (FP) detections.

In this study, we performed an indel calling evaluation with variant calling tools that represent different types of methods. We tested them with a variety of sequencing data types to discover the best indel calling size range and data type for each tool. This study uses both real sequencing data and simulated sequencing data. Simulated sequencing data can produce an accurate indel truth set, avoiding, as much as possible, any potential unmarked true positive (TP) indels in truth set which may be marked as FP results from the detections of the tools during evaluation. However, the production of simulated sequencing data, as *in silico* simulation processes, underestimates the potential technical errors in real-world sample preparation and sequencing steps [30]. Real-world sequencing data come from real experiments and are indisputably the ideal data for tool evaluation. However, to our knowledge, there are no real sequencing data coupled with a truth set that cover a wide range of variable-sized indels without technical bias, such as tools involving truth set generation, that would be suitable for our evaluation. Therefore, this study used a semi-simulated WGS dataset which were adopted from the HuRef genome data with four different sequencing settings to evaluate eight candidate tools with a wide size range of indels [31]. In addition, we also used the following three real-world sequencing datasets: Genome in a Bottle (GIAB) NA24385 WES data [32], CHM1 cell line WGS data [33], and targeted gene panel sequencing data to evaluate the performance of tools for indel calling. Together with all the selected datasets, our study aimed to deliver an unbiased and comprehensive evaluation of variant calling tools for indel calling.

## Materials and methods

### Ethics statement

The targeted gene panel sequencing dataset in this study was obtained from leukemia patients. The dataset was analyzed anonymously. The study involving human participants was reviewed and approved by the Ethics Committee of Turku University Hospital (approval no. 30/1802/2019) and Turku Clinical Research Centre (approval no. T012/014/19).

### Variant calling tools

For the evaluation, we selected the following eight widely used variant calling tools: DeepVariant [23], DELLY [21], FermiKit [34], GATK HaplotypeCaller (GATK HC) [16], Pindel [12], Platypus [17], Strelka2 [22], and VarScan [19], which all represent different underlying methods for indel calling (Table 1). Below, the main features of each tool are described:

**Table 1:** Methodological strategies of the tools included in this study.

Variants Calling tools	Paired-end reads	Split reads	Sequence assembly	Gapped sequence alignment	Machine learning
DeepVariant v0.7.1				X	X
DELLY v0.7.9	X	X			
FermiKit v0.13			X		
GATK HC v4.0.1.2			X	X	
Pindel v0.2.5.b9		X			
Platypus v0.8.1.1			X	X	
Strelka2 v2.9.2			X	X	X
VarScan v2.4.3				X	

#### DELLY

DELLY is a variant calling tool that is designed for SV discovery [21]. It uses aligned reads in a sorted, indexed, and duplicate-marked BAM file as input. It outputs a binary variant call file, which can easily be converted to a commonly used variant call format (VCF) [35,36]. DELLY integrates a paired-end reads method and a split-read method with data from different insert size libraries to call SVs at a single nucleotide resolution. DELLY can detect multiple types of SVs, such as deletions, tandem duplications, inversions, translocations, and medium-sized insertions, thus being able to call both large and medium-sized indels.

#### DeepVariant

DeepVariant is a variant calling software that makes use of a deep convolutional neural network [23]. It is implemented as an analysis pipeline that takes aligned reads in a BAM file as input with three steps of discovery and encoding of the candidate variants, genotyping using the neural network, and writing output in the VCF file. DeepVariant stacks so-called inception modules, which have concatenated multiple convolution filters. Upon processing, the output layer returns a probability distribution of genotypes for each candidate variant. The non-reference sites are then assigned the most likely genotype and written into a VCF file. DeepVariant supplies two trained models for WGS and WES data predictions. The WGS model was trained on NA12878/HG001 and NA24631/HG005 samples, and the WES model was trained on NA12878/HG001 and NA24631/HG005 samples in version 0.7, respectively.

#### FermiKit

FermiKit is a *de novo* sequence assembly-based variant calling pipeline for Illumina whole-genome germline data [34]. It takes FASTQ files as input, assembles reads into contigs, and then maps them against a reference genome. It calls SNVs, short indels, and SVs from the read alignment with error correction. FermiKit uses several built-in modules to perform error correction, *de novo* assembly and mapping and then parses the “pileup” output and extracts alignment break points to call SNVs, short indels and SVs in VCF format

#### GATK HaplotypeCaller

GATK HC is a variant calling tool that calls germline SNVs and indels via local re-assembly [16]. It first defines active regions from input BAM files that show differences with reference sequences. These active regions indicate the evidence of variants by using CIGAR information from BAM files such as mismatches, insertions, or deletions in the mapped reads and the high base-quality soft clip reads. Then it splits reads from active regions into k-mers to identify candidate haplotypes by re-assembling them with de-Bruijn-like graphs. After that, a pair Hidden Markov Model (pair-HMM) is built with state transition probabilities from the read base qualities to calculate the likelihood that each read was derived from each haplotype. The haplotype likelihood of each read from the pair-HMM is used to calculate raw genotype likelihoods using a Bayesian model. The genotype likelihoods are then used to call raw variants, which are output in a VCF format.

#### Pindel

Pindel is a split-read-based pattern growth algorithm [37] that can detect the breakpoints of large deletions and medium-sized insertions from BAM files of paired-end short read sequencing data and report variants in VCF format with single nucleotide resolution [13]. Pindel first extracts the paired-end sequencing reads from the mapping results, keeping the paired reads for which only one read can be mapped uniquely to the genome with no mismatch and the other read cannot be mapped to the reference genome under a certain alignment score. The 3′ end of the mapped read determines an anchor point. With the anchor point, a sub-region from the direction of the unmapped read will be searched with a user-defined maximum detection size. The 3′ and 5′ end fragments of the unmapped read will be used by the pattern growth algorithm to search for possible unique substrings on a reference genome within a certain size of a sub-region. The gap between the 3′ and 5′ end fragments of the unmapped read is reported as an indel if at least two complete reads can be assembled with possible substrings from both the 3′ and 5′ end fragments of unmapped reads.

#### Platypus

Platypus is a haplotype-based variant caller with local sequence assembly in a Bayesian statistical framework [17]. It uses BAM files as input and outputs VCF files. First, it constructs candidate variants from the CIGAR strings of the BAM files, local re-assembly, and external sources (VCF files provided by the user). Candidate variants are then assigned with pre-defined priors at the generation stage, and candidate haplotypes are generated. The likelihood of each haplotype is calculated with an HMM by aligning a read to the haplotype sequence. Then, an expectation-maximization algorithm is applied to estimate the haplotype frequency using a diploid genotype model. With the haplotype frequencies, the posterior supports for variants are calculated by comparing the likelihoods between all haplotypes, including haplotypes that do not include a particular variant. Variants whose posterior support exceeds a threshold and that pass a number of pre-defined filters are called.

#### Strelka2

Strelka2 is a variant calling method for small variants [22]. It takes a BAM file as input and outputs a VCF file, and it is designed for both germline and somatic sequencing applications. The germline mode workflow defines several parameters from sequencing data and applies an indel error model to estimate the error rates of indels and SNVs. It first defines active regions likely to have variants, and then uses alignments or local re-assembly of sequencing reads to generate candidate haplotypes and variant alleles of these active regions. Subsequently, candidate variants that passed the pre-defined filtering are phased and genotyped by reads re-alignment with a probability model. In the final step, Strelka2 applies pre-trained supervised random forest models for SNVs and indels, which are trained on the labelled data of the Platinum Genomes project [38] to improve variant calling precision.

#### VarScan

VarScan takes a single mpileup file (text pileup file from BAM files) as input, and outputs a VCF file [19]. It first scores and sorts the BAM file and discards reads that were mapped ambiguously to multiple positions or with a low identity as well as unmapped reads where the aligner failed to map anywhere in the genome. The uniquely mapped reads are used to detect variants and determine the total number of reads that support each unique variant. VarScan then filters each predicted variant by the overall coverage, number of supporting reads, p-value, variant allele frequency, base quality, and the number of strands that are observed in the predicted positions of the variants.

### Datasets

To comprehensively evaluate the variant calling tools, we used both simulated sequencing data and real sequencing data (Table 2). We created a semi-simulated dataset with four different sequencing settings to extract a wide size range of indels from a real human sample, with known indels available as truth sets. Additionally, we utilized three real datasets that represented different sequencing data types. The details of the datasets are described below.

**Table 2:** The semi-simulated WGS sequencing and real sequencing datasets used in this study.

Data	Read length	Coverage	Data content	Usage	Sequencing platform
Semi-simulated data	100bp	5×	WGS data (chr1, chr2)	Small and large indels	Illumina HiSeq 2000
	100bp	30×	WGS data (chr1, chr2)	Small and large indels	Illumina HiSeq 2000
	100bp	60×	WGS data (chr1, chr2)	Small and large indels	Illumina HiSeq 2000
	250bp	30×	WGS data (chr1, chr2)	Small and large indels	Illumina MiSeq v3
GIAB NA24385 data	126bp	135×	WES data	Small indels (<50bp)	Illumina HiSeq 2500
CHM1 cell line data	101bp	41×	WGS data	Large indels (≥50bp)	Illumina HiSeq PE-101
Targeted gene panel sequencing data	150bp	NextSeq: 2298× & MiSeq: 6651×	Targeted sequencing data	Clinical indels (3bp - 52bp)	Miseq v3 or NextSeq 500 v2

The National Center for Biotechnology Information recommends variants larger than 50bp to be submitted to dbVar, a database of human genomic structural variation, while variants less than 50bp should be submitted to dbSNP, a database containing human SNVs, small indels, and other types of small variants [39,40]. Based on this separation, small indel calls (< 50bp) and large indel calls (≥ 50bp) from the real sequencing data were evaluated separately.

#### The semi-simulated WGS dataset covering a wide size range of indels with varying coverages and read lengths

In order to simulate as realistic data as possible with precise and fair indels for benchmarking purposes, we utilized HuRef genome data [31]. The original HuRef dataset was generated by Sanger sequencing, and the variants were detected by Celera Assembler [31]. The use of Sanger sequencing may potentially limit mapping issues caused by short sequencing reads, and an independent variant calling method for our candidate tools makes these data very suitable here. HuRef data also allowed us to reproduce a realistic dataset that would capture the challenges of small indel and large indel calling in the human genome. If the indels were to be inserted randomly across the genome it would underestimate the proportion of indels located in ambiguous regions, where the indels may be represented in different positions [41] (S1 and S2 Figs). Here, we reconstructed chromosome 1 and chromosome 2 of the HuRef genome based on human reference genome hg19 via methods similar to [41,42] and inserted indels of a real human individual into the corresponding position in a reference genome (S1 File)⁠. In addition, we reconstructed the semi-simulated genome with two different haplotypes by randomly selecting variants from different size ranges and inserting them into only one of the haplotypes as heterozygous variants or into both haplotypes as homozygous variants. In total, 43,066 insertions and 45,223 deletions were included in our evaluation study (S1 Table).

We created simulated paired-end sequencing reads using the NGS read simulation tool ART [43] with three different coverages of 5×, 30× and 60×. The read length of the simulated paired-end sequencing data was 100bp⁠⁠. In addition, we created another simulated paired-end sequencing dataset with 30× coverage and 250bp read length to compare how read length would affect indel calling. For each dataset, the sequencing coverage was contributed by the two haplotypes with an approximate ratio of 1:1, making it representative of a naturally diploid sample (S1 File)⁠. The semi-simulated paired-end sequencing dataset is publicly available at https://doi.org/10.5281/zenodo.5774300.

#### Genome in a Bottle NA24385 WES dataset for small indels (< 50bp)

The GIAB NA24385 dataset was used to assess the small indel calling of tools from the real sequencing data. The GIAB datasets are considered as the gold standard among variant call sets. The high confidence in their variants is achieved by integrating and curating several call sets produced using competing sequencing platforms and variant calling tools. The variant datasets consist of seven individuals, of which one individual from the trio of Ashkenazi Jews (NA24385/HG002) was selected for this study [32]. The corresponding sequencing data were provided in a variety of conditions and technologies; the NA24385 WES data from Oslo University Hospital were selected for use in this study. Sequencing was performed on the Illumina HiSeq 2500 instrument, with the Agilent SureSelect Human All Exon V5 capture kit, and yielded 150bp paired-end reads. The raw 135× sequencing data for GIAB NA24385 was downloaded using the Sequence Read Archive (SRA) toolkit with accession SRX1453593 [44]. In total, 5,436 indels with sizes < 50bp were used to represent the human individual small indels set and evaluate the tools in this study (S3 Fig).

#### CHM1 cell line WGS dataset for large indels (≥ 50 bp)

The SV dataset of the CHM1 cell line was used to assess the large indel calling of tools from real sequencing data. The CHM1 cell line is a haploid human hydatidiform mole lacking allelic variation [33]. The SV dataset of the CHM1 cell line was produced by a single-molecule, real-time sequencing technology at a 54× sequencing coverage, which generated 18,467 indels between 50bp and 10,000bp that were used in our study (S4 Fig). All the sequence reads were aligned to GRCh37 using a modified version of the PacBio long read aligner and generated local assemblies by Celera and Quiver. Next, the SVs of the CHM1 cell line were characterized systematically by a custom computational pipeline. The NGS short reads sequencing data of the CHM1 cell line were produced on an Illumina HiSeq 2500 with 41× sequencing coverage and a 101bp read length of paired-end reads. The raw sequencing data of the CHM1 cell line from the Illumina platform was downloaded using the SRA toolkit with the accession SRX652547 [33].

#### Targeted gene panel sequencing dataset

A total of eight targeted NGS datasets from patients with either acute myeloid leukemia (AML), essential thrombocythemia (ET), or myelofibrosis (MF) were collected from Turku University Hospital (TYKS), Finland (S2 Table). A targeted amplicon-based panel, the TruSight Myeloid Sequencing panel (Illumina, US), was used to detect variants in diagnostic samples. The myeloid gene panel targets 54 genes with 568 amplicons, ranging from 225bp to 275bp in length. The combined coverage for libraries was 141 kb. For library preparation, 50 ng of DNA per sample was used, and the Illumina protocol was followed. The samples were pooled in series of 8 to 24 to obtain sufficient sequencing depth and 2 × 150 cycle sequencing runs were performed on the Illumina platform (Miseq v3 chemistry or NextSeq 500 v2 mid output chemistry). To analyze the quality of the sequencing run, the parameters and data output from each run were compared against the specifications outlined by the manufacturer (Illumina), as follows: cluster density (1200–1400 K/mm2 and 170–220 K/mm2 for MiSeq and NextSeq, respectively); Q30 greater than 75% in both systems; and the total number of reads passing the filter and the total data yield of the run were evaluated to approve the data of the run. The average coverage of the amplicons in the data generated with the NextSeq 500 platform was 22987× and for the MiSeq 6651×. As a truth set we used the somatic indels in genes *CALR* and *CEBPA* that were determined for diagnostic purposes in the Laboratory of Molecular Hematology and Pathology, TYKS Laboratory Division, Turku, Finland. The 52bp deletions in *CALR* exon 9 were analyzed with capillary electrophoresis after PCR amplification, as described by [45]. The *CEBPA* indels ranging from 3bp to 36bp were determined as part of the diagnostic testing in Labor für Leukämiediagnostik, Munich, as described by [46].

### Evaluation criteria

We evaluated variant calling tools using their default running parameters, with some exceptions (S1 File). For data pre-processing such as sequencing reads alignment, we built a fixed bioinformatics pipeline to deal with both semi-simulated and real sequencing data (S1 File).

For the evaluation of indel calling with the semi-simulated WGS dataset, we first filtered out all the SNVs from each tool’s output, using vcftools [36] and only kept the indel results for further evaluation. We considered a tool-detected indel call as a TP if 1) (position-match) the indel position deviation of a tool-detected indel and a truth-indel was between ±10% of the truth-indel size (the upper limit for position deviation was 50 bp); 2) (size-match) the size difference between a tool-detected indel and a truth-indel was < 25% of the size of the truth-indel; 3) (genotype-match) the genotypes of a tool-detected indel and a truth-indel were consistent. The tool-detected indels that failed to give genotype information were considered as FPs. In our truth set, there were no multiallelic indels; if a tool called an indel as a multi-allelic variant, the record was split as multiple records for each allele.

For the GIAB NA24385 WES datasets, we used hap.py (https://github.com/Illumina/hap.py) to assess the evaluation results of each tool (S1 File). Hap.py is a VCF file comparison tool for diploid samples, that can benchmark the variant calling results made by a variant calling tool against a truth set [47].

For the CHM1 cell line WGS datasets, we defined a TP as a tool-detected, filter-passed indel that has at least 20% overlap with a truth-indel via BEDtools [48] (S1 File). The criteria were kept loose because previous studies [41,49] have reported that the variant calling methodology for CHM1 cell line WGS datasets has its own bias, which leads to a low concordance between tool-detected indels and truth-indels (S1 File).

The performance of tools on the semi-simulated WGS dataset and GIAB NA24385 WES datasets was evaluated by calculating precision rate, recall rate, and F1 score. The performance of the tools on the CHM1 cell line WGS datasets was evaluated by calculating false discovery rate (FDR) and sensitivity (recall). For clinical targeted gene panel sequencing data, due to the limited number of indels validated by clinical methods, we manually checked the results of each tool and summarized the results. For the semi-simulated WGS dataset and the CHM1 cell line WGS dataset, the indel calling was evaluated for different indel size ranges as right half-open intervals.


$$Precision=\frac{TP}{TP+FP}$$ (1)

$$Recall=\frac{TP}{TP+FN}$$ (2)

$$F1\;Score=2*\frac{Precision*Recall}{Precision+Recall}$$ (3)

$$FDR=\frac{FP}{TP+FP}$$ (4)

## Results

### Evaluation of small indel (< 50bp) and large indel (≥ 50bp) calling using the semi-simulated WGS dataset

**Fig 1:**
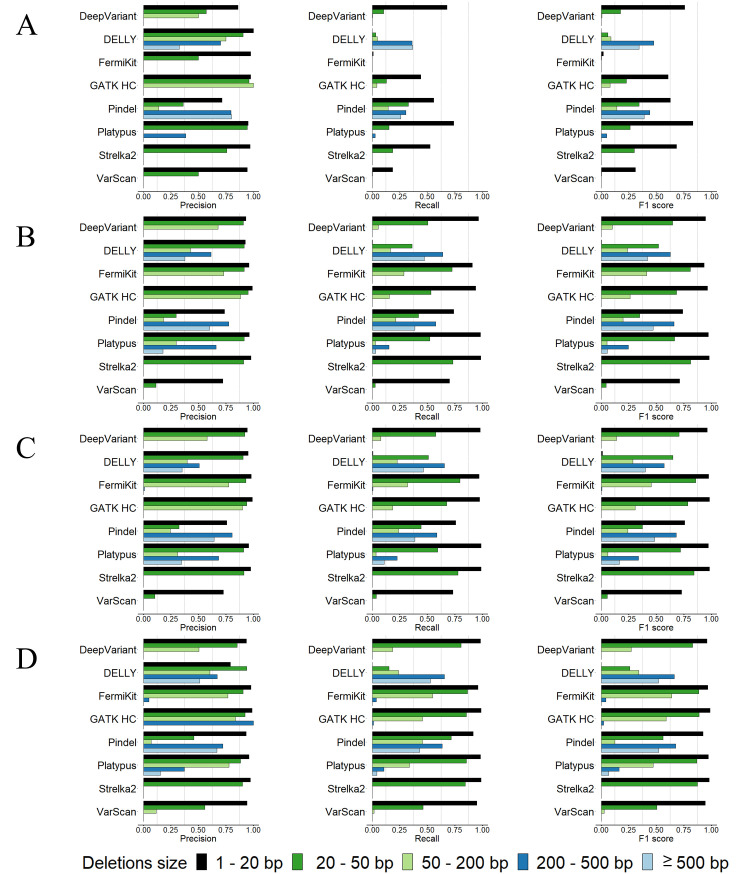
Precision rates, recall rates and F1 scores for tools’ deletion calling using the semi-simulated datasets. (A) 5× coverage, 100bp read length sequencing data. (B) 30× coverage, 100bp read length sequencing data. (C) 60× coverage, 100bp read length sequencing data. (D) 30× coverage, 250bp read length sequencing data.

**Fig 2:**
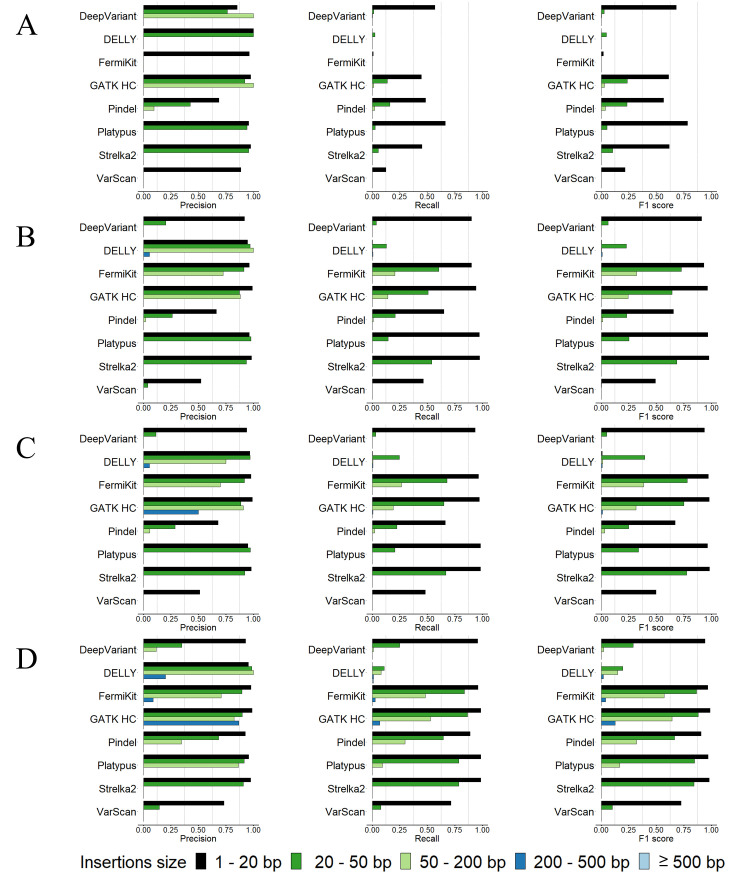
Precision rates, recall rates and F1 scores for tools’ insertion calling using the semi-simulated datasets. (A) 5× coverage, 100bp read length sequencing data. (B) 30× coverage, 100bp read length sequencing data. (C) 60× coverage, 100bp read length sequencing data. (D) 30× coverage, 250bp read length sequencing data.

We evaluated the tool-detected indels of each tool for different sequencing coverages and read lengths with the semi-simulated dataset in five indel size ranges of 1bp – 20bp, 20bp – 50bp, 50bp – 200bp, 200bp – 500bp, and ≥ 500bp (Figs 1 and 2 and S3 Table).

Although having good precision rates in general, the majority of tools had relatively low recall rates with the 5× coverage sequencing data, especially FermiKit, whose recall rates of all indel size ranges were below 0.01 (Figs 1A and 2A). With increasing sequencing coverage, the recall rates and the F1 scores of the tools were improved, and the improvement from 5× to 30× was more obvious than the improvement from 30× to 60× (Figs 1 and 2). Some very low recall rates made precision rates fluctuate greatly, but overall, the precision rates of the tools showed less differences than the recall rates between coverages (Figs 1 and 2). FermiKit benefited the most with increasing coverages, which indicated it cannot work well with low coverage sequencing data. With the 5× coverage sequencing data, the F1 scores of DeepVariant, GATK HC, Pindel, Platypus, and Strelka2 for the indels in size range 1bp – 20bp were over 0.5, which indicated that for gapped sequence alignment-based, split-read-based and machine learning-based indel calling algorithms, the detections of indels smaller than 20bp might be more effective than indels larger than 20bp with low coverage sequencing data (Figs 1A and 2A). DELLY and Pindel had F1 scores around 0.4 for deletions in size ranges 200bp – 500bp and ≥ 500bp with the 5× coverage sequencing data, which showed that DELLY and Pindel had relatively good abilities to call large deletion with low coverage sequencing data. In general, Pindel had the best performance with the 5× coverage sequencing data. From the F1 scores, the results demonstrated that a higher sequencing coverage generates a better indel calling result (Figs 1 and 2).

For the assessment of how different read lengths affect indel calling, we evaluated the performance of tools by using the 100bp read length and the 250bp read length sequencing data with 30× coverage (Figs 1 and 2). We observed that the variant calling tools had wider detection ranges and better precision rates, recall rates and F1 scores with the 250bp read length sequencing data than with the 100bp read length sequencing data with the same 30× coverage. The indel callings in size range ≥ 50bp were remarkably improved. In general, compared with 30× coverage, 100bp read length sequencing data, the improvement of the tools’ performance with 30× coverage, 250bp read length sequencing data were slightly better than the improvement of the tools’ performance with 60× coverage, 100bp read length sequencing data (Figs 1B, 1C, 1D, 2B, 2C and 2D). The results indicated that above a certain sequencing coverage, indel calling may benefit more by the increased read length than the increased coverage of sequencing data. Although having low recall rates, DeepVariant, FermiKit, GATK HC, Platypus and VarScan detected larger indels that they were not able to detect with 100bp read length sequencing data.

From our results, we can see that tools with different algorithms showed different best variant calling size ranges. Taking the 30× coverage, 100bp read length sequencing data as an example, for deletions in size range 1bp –20bp (Fig 1), DeepVariant, FermiKit, GATK HC, Platypus, and Strelka2 had both precision rates and recall rates over 0.9. The precision rates and recall rates of Pindel and VarScan for deletions in size range 1bp – 20bp were around 0.7. DELLY only detected 98 TP deletions in size range 1bp – 20bp. For deletions in size range 20bp – 50bp, the recall rates decreased remarkably for the majority of the tools, while DELLY started to detect deletions in this size range but with a low recall rate. FermiKit and Strelka2 were the only two tools that had recall rates over 0.7 in this deletion size range. Except for Pindel and VarScan whose precision rates were low, the other tools all had precision rates over 0.9 for deletions in this size range. For deletions in size range 50bp – 200bp, a decrease was seen in both precision rates and recall rates for all the tools. FermiKit had the best F1 score in this deletion size range, being little over 0.4. Strelka2 and VarScan failed to detect any deletions longer than 50bp. DELLY, Pindel, and Platypus were the only tools that detected deletions in size ranges 200bp – 500bp and ≥ 500bp; Pindel had the best precision rate, and DELLY had the best recall rate. Platypus had relatively good precision rates to detect deletions in this size range, but the recall rates were relatively low. For insertion calling with the 30× coverage, 100bp read length sequencing data (Fig 2), DeepVariant, FermiKit, GATK HC, Platypus, and Strelka2 had precision rates and recall rates around 0.9 for insertions in size range 1bp – 20bp. Pindel and VarScan had relatively low precision rates and recall rates, while DELLY only detected 69 TP insertions in this size range. For insertions in size range 20bp – 50bp, a notable decrease in recall rates was observed. Only FermiKit, GATK HC, and Strelka2 had recall rates over 0.5. DELLY and Platypus had high precision rates but low recall rates for detecting insertions in this size range, while DeepVariant and Pindel had both low precision rates and recall rates. VarScan had a very low recall rate and only detected one TP insertion in this size range. For insertions in size range 50bp – 200bp, all the tools performed poorly. Only FermiKit and GATK HC had recall rates around 0.15, and all the other tools either had very low recall rates or even failed to detect insertions in this size range. For insertions ≥ 200bp, only DELLY detected one TP insertion, while the other tools failed to detect any TP insertions in this size range. In general, the tools performed better on deletion calling than insertion calling.

**Fig 3:**
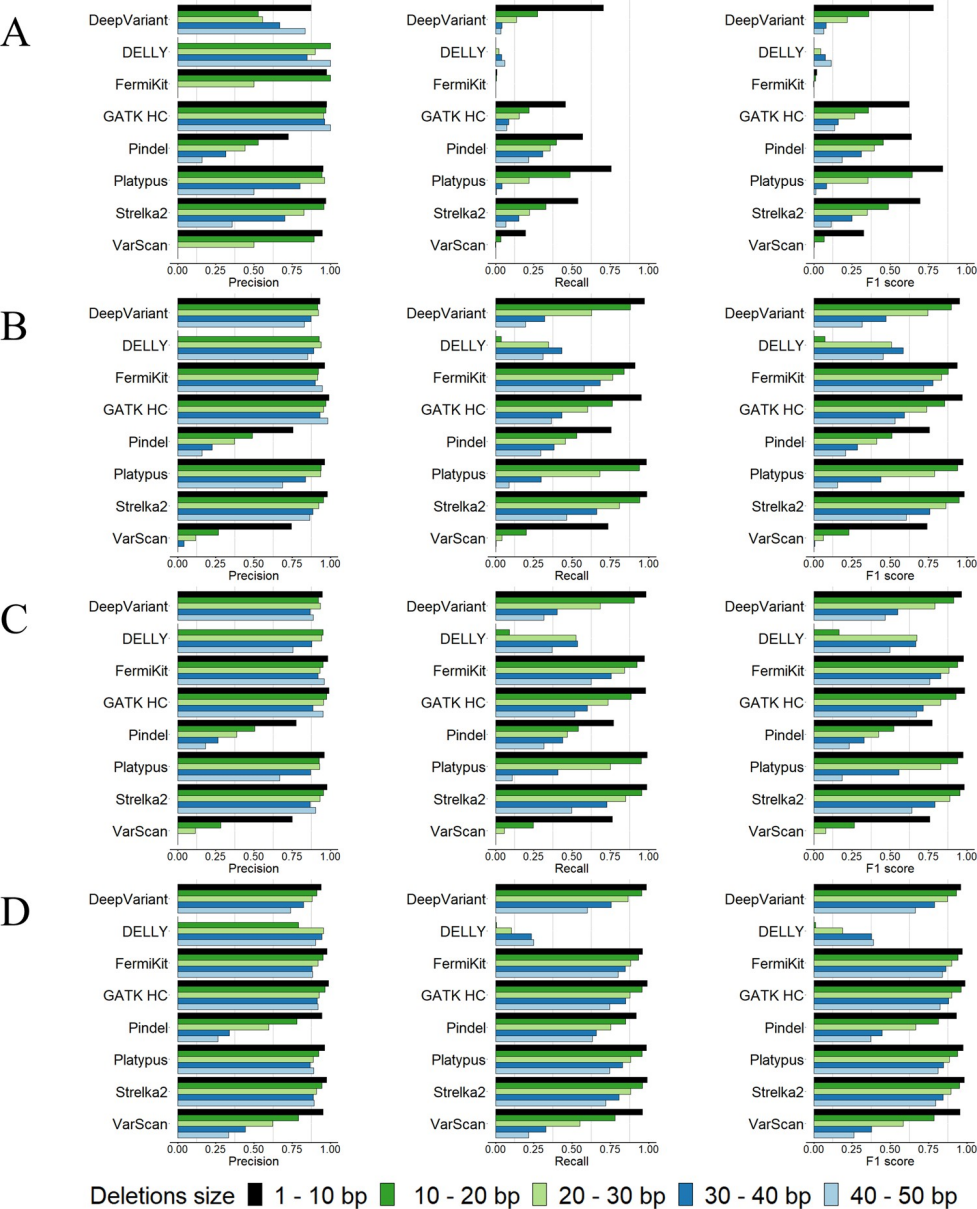
Precision rates, recall rates and F1 scores of the tools for calling deletions < 50bp using the semi-simulated datasets. (A) 5× coverage, 100bp read length sequencing data. (B) 30× coverage, 100bp read length sequencing data. (C) 60× coverage, 100bp read length sequencing data. (D) 30× coverage, 250bp read length sequencing data.

**Fig 4:**
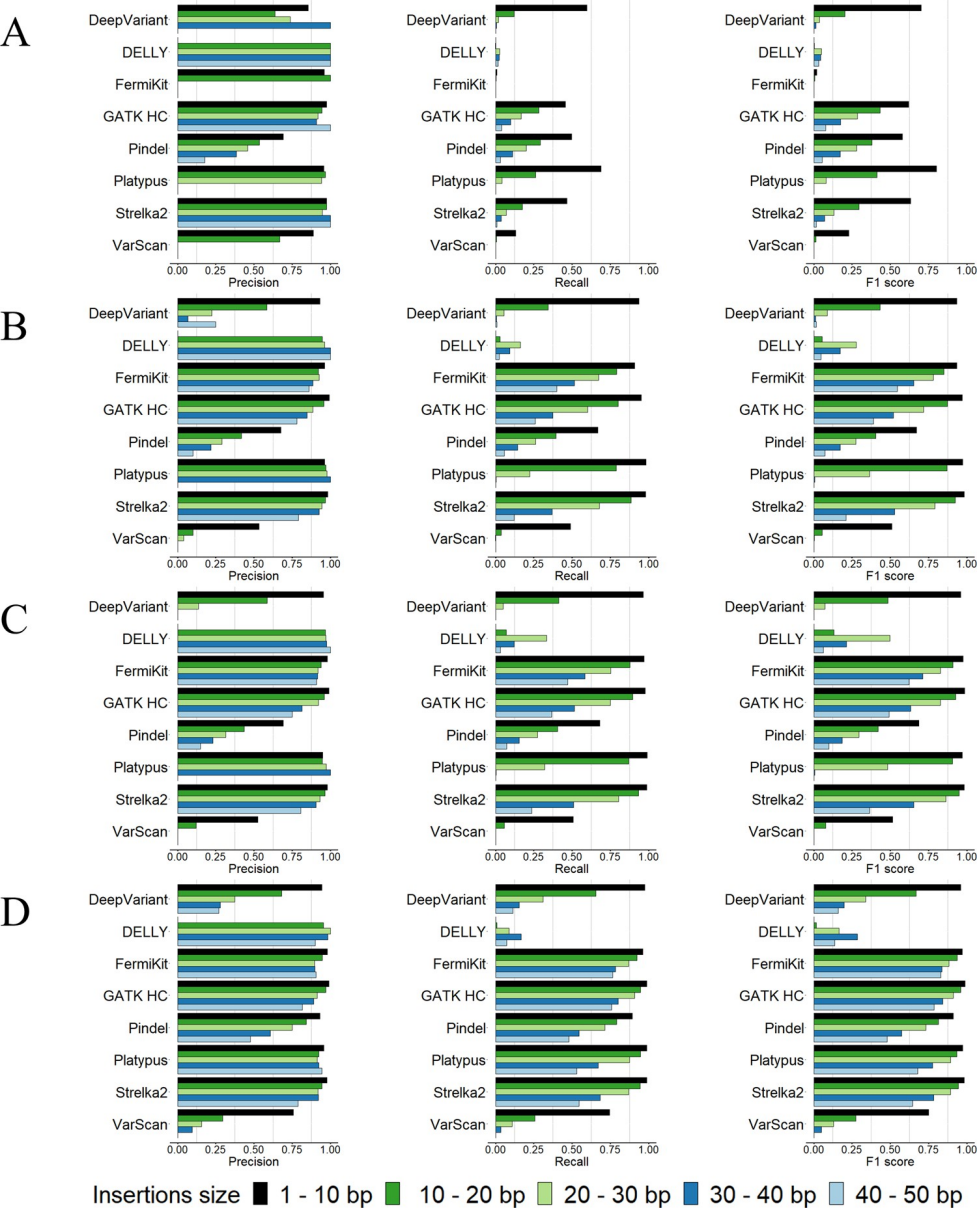
Precision rates, recall rates and F1 scores of the tools for calling insertions < 50bp using the semi-simulated datasets. (A) 5× coverage, 100bp read length sequencing data. (B) 30× coverage, 100bp read length sequencing data. (C) 60× coverage, 100bp read length sequencing data. (D) 30× coverage, 250bp read length sequencing data.

Previous research [50–53] has defined 50bp as the size border for SVs and small variants. We observed, however, that within the 1bp – 50bp range, the indel calling results were not consistent (Figs 3 and 4). For all the tools, there were notable decreases in the precision and recall rates with the increases in indel sizes, except for DELLY, which failed to call indels in the size range of 1bp -10bp. We further evaluated the size of tool-detected indels from DELLY and found that DELLY only called indels ≥ 15bp. Precision rates showed less decrease than recall rates, except for Pindel and VarScan, whose precision rates decreased remarkably. For indels in size ranges 1bp – 50bp, compared with 30× coverage, 100bp read length sequencing data, the improvement of the tools’ performance with 30× coverage, 250bp read length sequencing data were slightly better than the improvement of the tools’ performance with 60× coverage, 100bp read length sequencing data (Figs 3B, 3C, 3D, 4B, 4C and 4D). With indels < 50bp, we observed that the performance of the tools on deletion calling was better than insertion calling. In addition, we assessed the indel calling abilities of tools for medium size indels (30bp – 70bp) which were not studied by previous research (S5 Fig). The results suggested that for medium size indels, high coverage sequencing data is recommended. FermiKit and GATK HC were the best two tools to call indels in this size range. Strelka2 also had a good precision, rate, recall rate and F1 score, but it could not call any indel longer than 50bp. In general, compared with higher sequencing coverages, the longer read length sequencing data provided better precision rates and recall rates for longer indels sizes.

To further evaluate indel callings without genotypes taken into account, we relaxed our evaluation criteria to include indels that passed both position-match and size-match but that may have failed genotype-match (S6–S9 Figs). Without considering genotype-match, the precision rates and the recall rates of the tools were improved, especially for FermiKit, Pindel and VarScan. Although FermiKit still failed with 5× coverage sequencing data, it was able to call indels ≥ 200bp and became the best tool to call insertions ≥ 200bp with other semi-simulated sequencing data. The precision rates and the recall rates of Pindel and VarScan were improved with all settings of the semi-simulated datasets, which indicated that Pindel and VarScan detected many indels with correct positions and sizes but failed to detect indels with correct genotypes.

**Fig 5:**
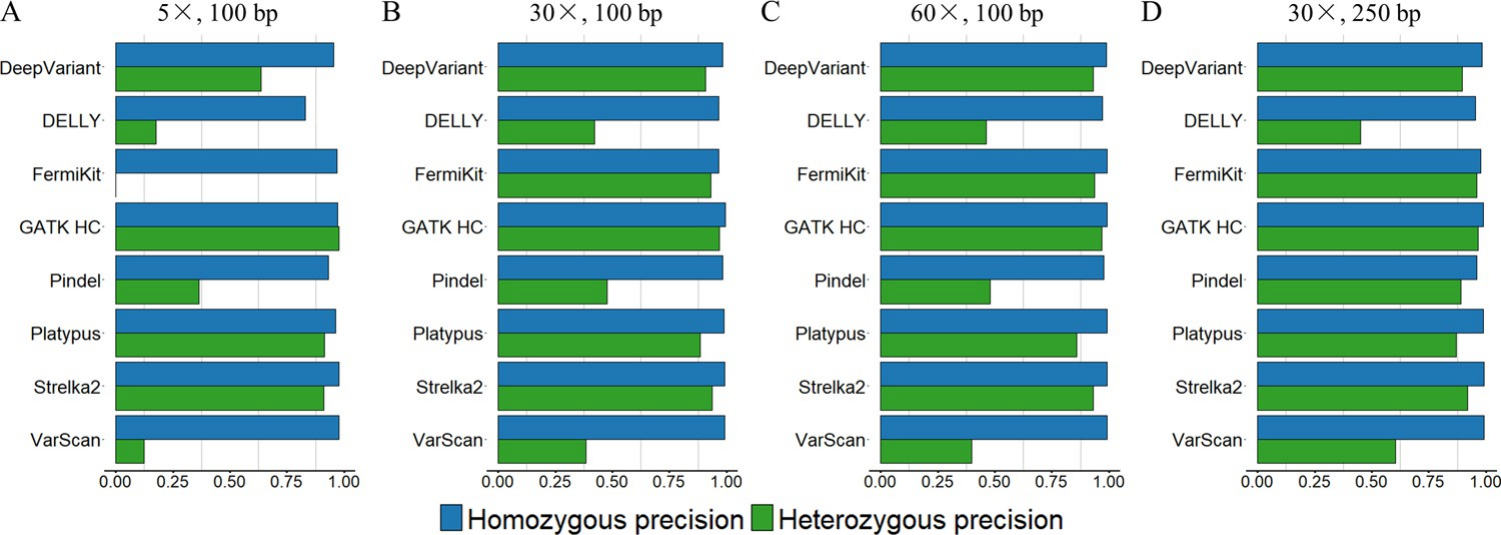
Homozygous and heterozygous precisions of variant calling tools using the semi-simulated dataset. (A) 5× coverage, 100bp read length sequencing data. (B) 30× coverage, 100bp read length sequencing data. (C) 60× coverage, 100bp read length sequencing data. (D) 30× coverage, 250bp read length sequencing data.

The genotype information of variants is needed to make reliable interpretation in the clinical setting. To further evaluate the performance of the tools with genotyping, we calculated the homozygous and the heterozygous precisions with the semi-simulated WGS dataset (Fig 5). In general, among the semi-simulated WGS dataset, the homozygous precision rates of tools were higher than the heterozygous precision rates. The homozygous calls of the tools all had good precision rates while the heterozygous calls of the tools showed differences. GATK HC had the best heterozygous precision rates while VarScan had the worst heterozygous precision rates among the different datasets, except FermiKit which failed with 5× coverage sequencing data. DeepVariant, DELLY, Pindel and VarScan had notable increase in heterozygous precision rates with increased sequencing coverage. Pindel and VarScan also had an increase in heterozygous precision rates with increased sequencing read length. Genotyping precisions of small indels (S10 Fig) are higher than those of large indels (S11 Fig). We also calculated the number of tool-detected indels marked as multiallelic indels or indels with non-valid genotype (S4 Table). Some of these indels passed the position-match and size-match but failed with the genotype-match. Due to the complexity of the human genome sequence, different tools may have different representations of the same indel, and supporting reads around indel breakpoints may not be adequate, thus tools may make a mistake for indel genotyping. DeepVariant, FermiKit, GATK HC, Platypus, and Strelka2 had few multiallelic indels. Although DELLY, Pindel, and VarScan had no tool-detected multiallelic indels, the reason might be that tools could only give genotype information as either homozygous, heterozygous, or even non-valid genotypes. In practice, it may limit for genotyping complex human variants. For indels with non-valid genotypes, DeepVariant, DELLY, FermiKit, Pindel, and Platypus had several such indels, while GATK HC, Strelka2, and VarScan had no such indels. GATK HC had less tool-detected multiallelic indels than Strelka2 and better indel calling performance than VarScan. In general, GATK HC was the best tool for genotyping indels.

**Fig 6:**
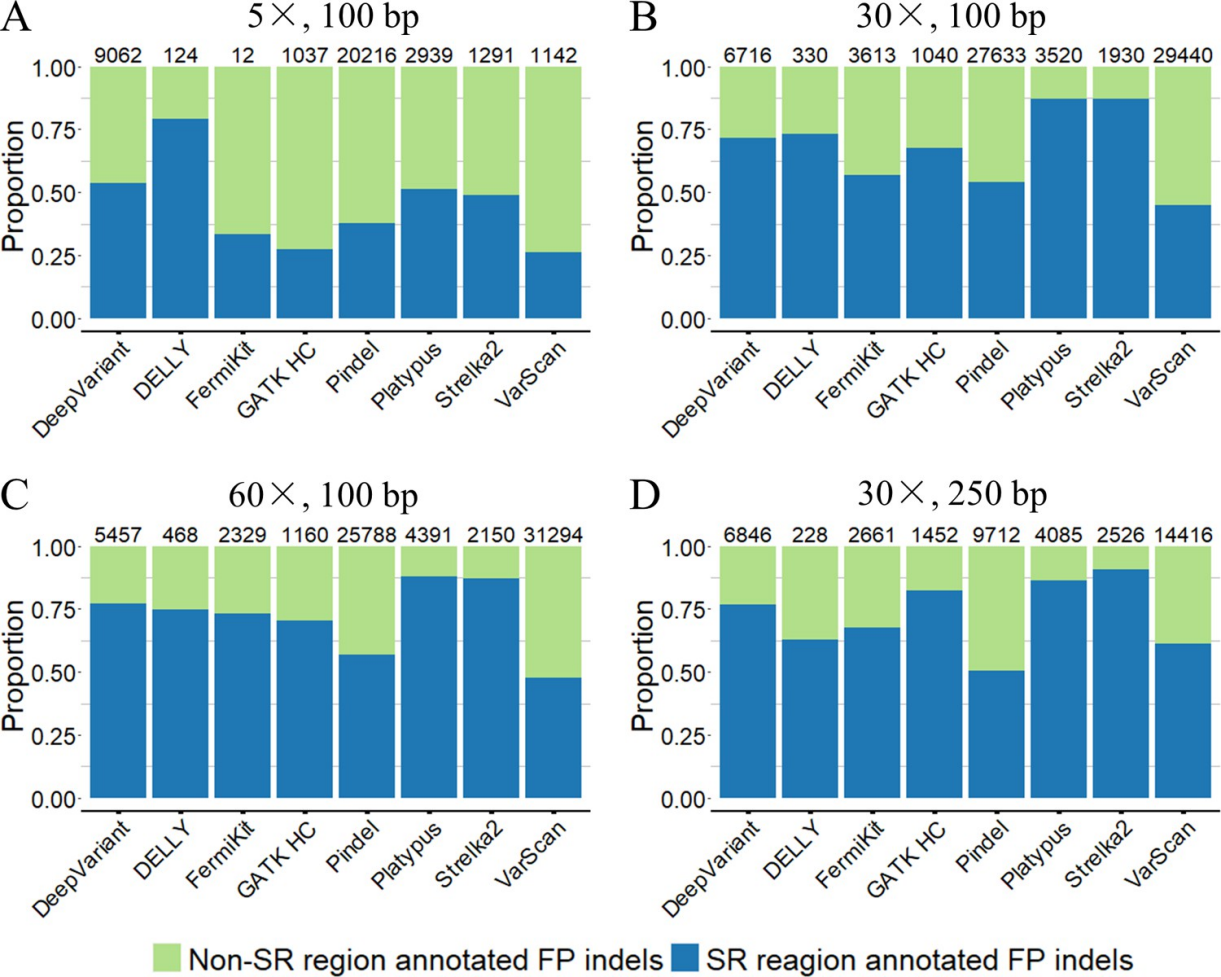
The proportions of SR regions annotated FP indels of the tools with the semi-simulated dataset. (A) 5× coverage, 100bp read length sequencing data. (B) 30× coverage, 100bp read length sequencing data. (C) 60× coverage, 100bp read length sequencing data. (D) 30× coverage, 250bp read length sequencing data. The numbers on the top of each bar are the total numbers of FP results called by each tool with corresponding sequencing data.

To better understand how the complexity of the genome sequence influences the FP results, we gathered the FP results from the semi-simulated WGS dataset and annotated the FP indel calls based on their locations by using the “Simple Repeats” (SR) track from the UCSC genome browser via BEDtools. The results showed that indels located in SR regions contain a large proportion of FP results, indicating that the breakpoint ambiguity caused by an SR region is the main challenge for indel calling (Fig 6). For 5× coverage sequencing data, the low read depth in indel breakpoints might be the main reason for the FPs. SR regions in the genome may cause a misalignment of reads and lead to further inaccurate indel calling, such as wrong positions or sizes due to the breakpoint ambiguity [41]. The breakpoint ambiguity of indels in SR regions may also cause incorrect allele frequency counting, thus leading to false genotype-level results (S12 Fig).

### Evaluation of small indel calling using GIAB NA24385 WES data

**Fig 7:**
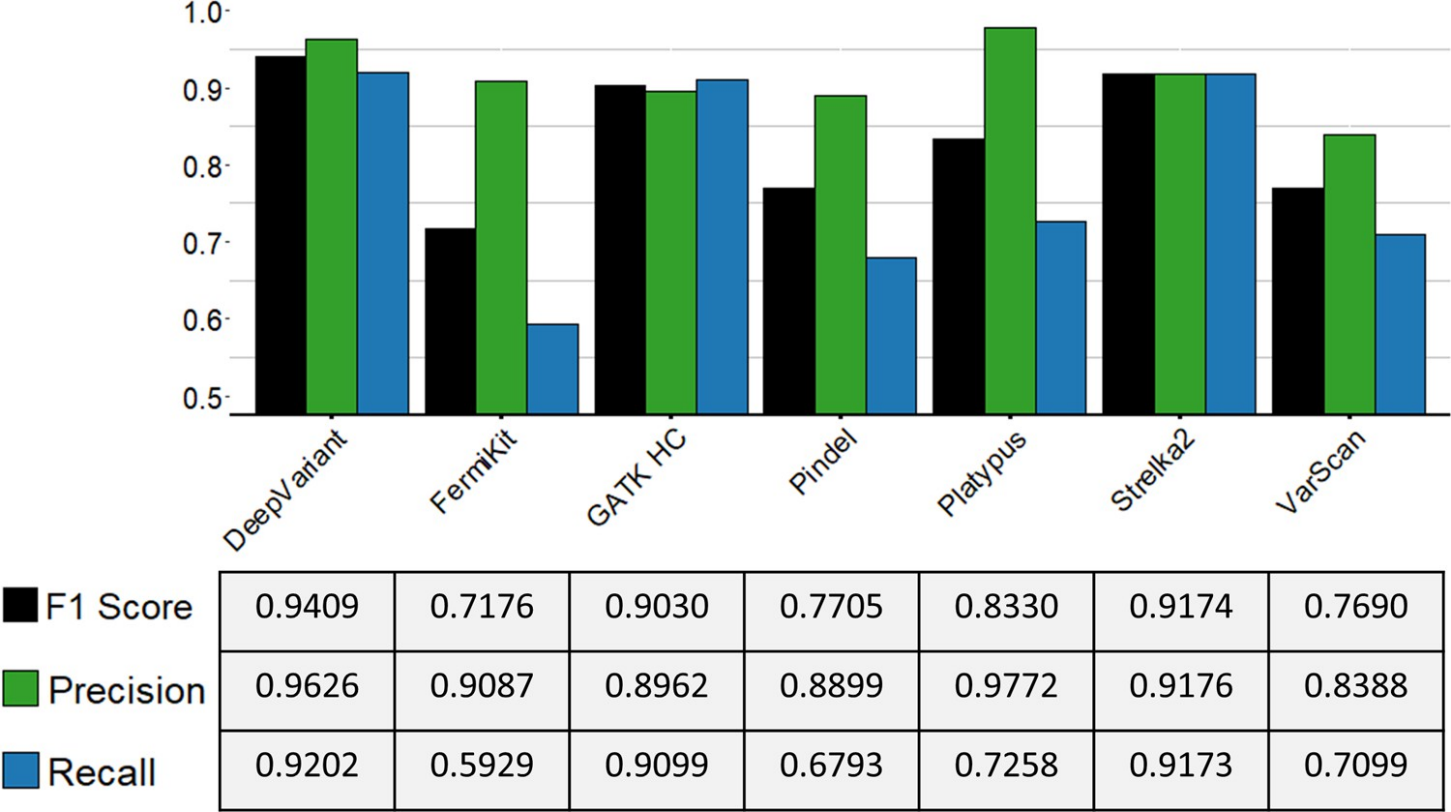
Indel calling evaluation results for variant calling tools with GIAB NA24385 WES data. The evaluation results were calculated using hap.py. The table below shows the values of the precision rates, recall rates, and F1 scores of each tool.

From the F1 scores of the tools with the semi-simulated WGS dataset, we selected the tools that were suitable for small indel calling (DeepVariant, FermiKit, GATK HC, Pindel, Platypus, Strelka2, and VarScan) for further evaluation with real sequencing data. The results in the GIAB NA24385 WES data (Fig 7) from hap.py showed that DeepVariant had the best recall rate and that DeepVariant, Strelka2, and GATK HC were the only tools with recall rates > 0.9. Platypus had the best precision rate, followed by DeepVariant, both of which had precision rates > 0.95. DeepVariant also had the best F1 score, followed by Strelka2, and GATK HC each had an F1 score > 0.9. At the genotype-level prediction, VarScan performed worse than the other tools, and Strelka2 had the best performance for genotyping (S5 Table). To our surprise, FermiKit, which is primarily designed for WGS data, still had a relatively fair performance, with a precision rate > 0.9 and a recall rate nearly 0.6. DeepVariant and Strelka2 had higher F1 scores than the other tools with the GIAB NA24385 WES data, which may indicate that machine learning tools have advantages of indel calling abilities over other indel callers.

### Evaluation of large indel calling using CHM1 cell line WGS data

**Fig 8:**
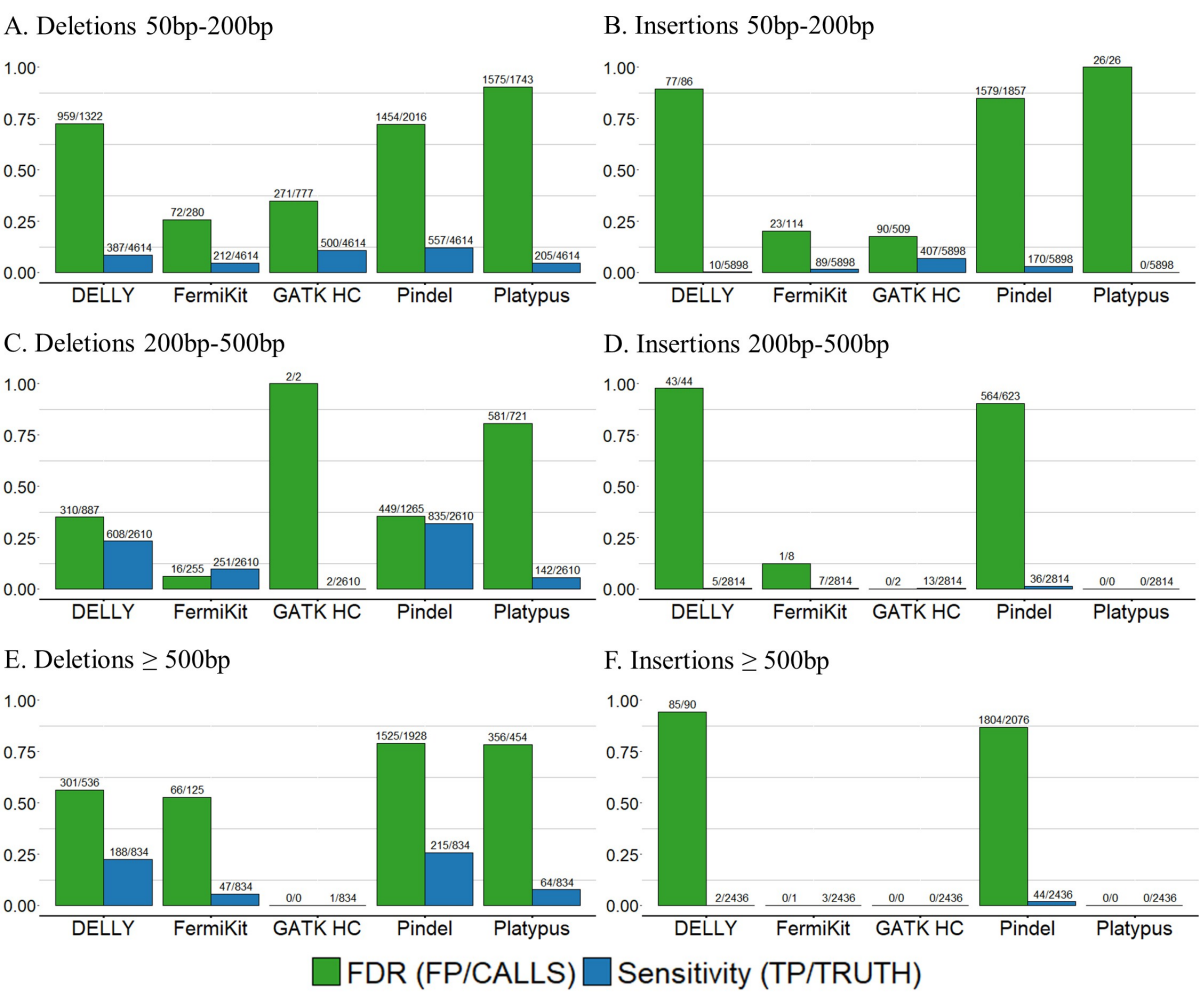
Indel calling results for variant calling tools with CHM1 cell line sequencing data. (A) The FDRs and sensitivities of deletion calls in size range 50bp – 200bp. (B) The FDRs and sensitivities of insertion calls in size range 50bp – 200bp. (C) The FDRs and sensitivities of deletion calls in size range 200bp – 500bp. (D) The FDRs and sensitivities of insertion calls in size range 200bp – 500bp. (E) The FDRs and sensitivities of deletion calls in size range ≥ 500bp. (F) The FDRs and sensitivities of insertion calls in size range ≥ 500bp. The number of FPs and the number tool-detected indels are listed above FDRs. The number of TPs and the number of indels in truth set are listed above sensitivities.

Because the CHM1 cell line is a human haploid hydatidiform mole, we chose the tools which had ability to call indels ≥ 50bp based on the position-match and size-match results of the semi-simulated WGS dataset (DELLY, FermiKit, GATK HC, Platypus, and Pindel) and further evaluated them with real sequencing data. We used our pre-defined criteria to evaluate the performance of the tools on large indels of size ranges 50bp - 200bp, 200bp - 500bp and ≥ 500bp in the CHM1 cell line WGS data (Fig 8 and S6 Table). For deletions in size range 50bp – 200bp, FermiKit had the lowest FDR 0.26, together with GATK HC which FDR was 0.35, were the only two tools with FDR below 0.5. Pindel and GATK HC were the only two tools which had sensitivity high than 0.1, even though the best sensitivity from Pindel was just 0.12. For deletions in size range 200bp – 500bp, FermiKit had the lowest FDR (0.06) but the sensitivity was only around 0.1 (0.09). DELLY and Pindel had similar FDRs, both around 0.35, but the sensitivity of Pindel was the best (0.32). For deletions ≥ 500bp, Pindel had the best sensitivity (0.26), followed by DELLY (0.23), but FDRs for all five tools were not good. For insertions, all the tools performed badly, especially for insertions ≥ 200bp. For insertions in size range 50bp – 200bp, only FermiKit and GATK HC had FDRs lower than 0.5, but the best sensitivity was GATK HC still lower than 0.1 (0.07). Although the overall variant calling of the tools only called limited numbers of indels, the performance of the tools on deletion calling was generally better than on insertion calling, and the results with indels in size range 50bp - 200bp were generally better than the results with indels in size range ≥ 200bp. Although GATK HC was designed for small variant calling, but the results showed GATK HC had the best performance with indels in size range 50bp – 200bp. Platypus had the worst performance and only detected a limited number of deletions. Even though the criteria were loose, the concordance of the predicted results from the tools and the CHM1 truth set was still remarkably low. A possible reason for this is that variants in repeat regions can be called by CHM1 cell line PacBio calls, but cannot be detected efficiently by Illumina short-read sequencing [49]. A previous study that used a CHM1 cell line dataset as a truth set also suggested that the truth set might be missing variants [41].

### Evaluation of indel calling using targeted gene panel sequencing dataset

We used amplicon-based clinical targeted gene panel sequencing dataset to further study the indel calling ability of the tools to call indels ranging from 3bp to 52bp in a real-world clinical cancer study (Table 3). The blood samples of leukemia patients were analyzed, where normal tissue was not available. However, indels have been shown to have clinical relevance in leukemia and such known, relatively common indels were included in this evaluation. Here, we only used the germline mode of the variant calling tools and conducted indel calling in a non-matched variant calling manner [54]. FermiKit is not designed for targeted sequencing data analysis, so it was not included in this evaluation. The results show that despite the fact that no tool detected the 22bp deletion of the *CEBPA* gene from sample 1 due to either misalignment of sequencing reads or low allele fraction, DeepVariant, GATK HC, Pindel and Platypus successfully detected all the rest of the known variants. DELLY and VarScan failed to detect two 3bp deletions of the *CEBPA* gene, and VarScan was the only tool that failed to detect one 36bp deletion of the *CEBPA* gene. Strelka2 was the only tool that failed to detect four 52bp deletions of the *CALR* gene.

**Table 3:** Indel calling results for the selected tools with targeted gene panel sequencing dataset of leukaemia patients.

Samples	Genes	Variants	DeepVariant	DELLY	GATK HC	Pindel	Platypus	Strelka2	VarScan
Sample 1	*CEBPA*	3bp insertion	Y	N	Y	Y	Y	Y	N
	*CEBPA*	22bp deletion	N	N	N	N	N	N	N
Sample 2	*CALR*	52bp deletion	Y	Y	Y	Y	Y	N	Y
Sample 3	*CALR*	52bp deletion	Y	Y	Y	Y	Y	N	Y
Sample 4	*CALR*	52bp deletion	Y	Y	Y	Y	Y	N	Y
Sample 5	*CALR*	52bp deletion	Y	Y	Y	Y	Y	N	Y
Sample 6	*CEBPA*	24bp insertion	Y	Y	Y	Y	Y	Y	Y
Sample 7	*CEBPA*	36bp deletion	Y	Y	Y	Y	Y	Y	N
	*CEBPA*	25bp deletion	Y	Y	Y	Y	Y	Y	Y
Sample 8	*CEBPA*	3bp insertion	Y	N	Y	Y	Y	Y	N

For each sample there are one or more indel varying from 3bp to 52bp either in *CEBPA* or *CALR*. We manually evaluated the variant calling tools’ results with our truth set. A successful call is marked with "Y", a failed call is marked with "N".

## Computational costs

**Fig 9:**
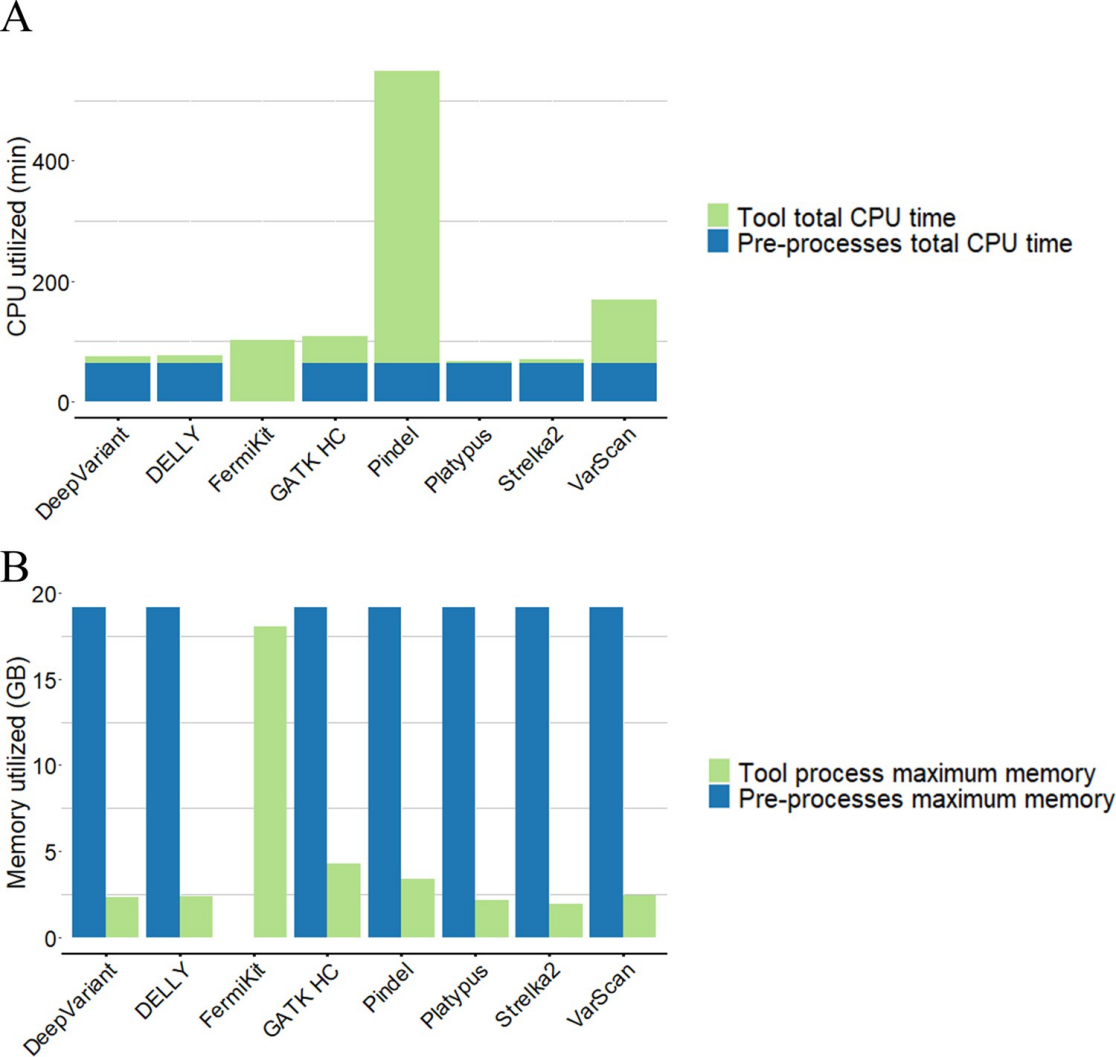
The running times and the maximum memory usages of variant calling tools. A) Total CPU times of each variant calling tool with 30× coverage, 250bp read length semi-simulated sequencing data. The total CPU time of pre-processing included aligning the sequencing reads into the BAM file. Tool total CPU time included analyzing the input BAM file into the output VCF format result. (B) Maximum memory usage of each variant calling tool with 30× coverage, 250bp read length semi-simulated sequencing data. Maximum memory of pre-processing included aligning the sequencing reads into the BAM file. Tool maximum memory usage included analyzing the input BAM file into the output VCF format result. Because FermiKit is a *de novo* assembly algorithm-based variant calling tool, which took sequencing reads as input, it did not require pre-processing.

We measured the running times and maximum memory usages of tools, based on one of the semi-simulated WGS data with 30× coverage and 250bp read length (Fig 9). All the indel calling processes, including read alignment and variant calling, were performed on a computer cluster managed by the free, open-source Simple Linux Utility for Resource Management. For each task we assigned 24 cores (2 GHz AMD EPYC Processor), with 32G memory on the computer cluster.

To save time and fully use the high-performance computational resources, parallelization is an important option for variant calling tools. Tools that are designed with a parallelization option are able to assign multiple CPUs to some steps of the data processing (DeepVariant, FermiKit, GATK HC, Pindel, Platypus and Strelka2). Tools without a parallelization option can only utilize one CPU to process the data (DELLY and VarScan). From the results (Fig 9), Platypus was the fastest tool, while Pindel was the slowest. Platypus used the least memory, while FermiKit required the most. The high memory consumption of FermiKit is explained by the reads alignment step that is included in the tool execution. The total utilized memory of FermiKit was lower than the memory consumption of the data pre-processing which produced the sorted and indexed BAM files as input for the other tools.

## Discussion

In this study, we evaluated eight variant calling tools for the indel calling of different indel size ranges from different types of NGS data. The tools represented different underlying algorithms including paired-end reads, split-read, *de novo* sequence assembly, gapped sequence alignment, and/or machine learning-based methods. The tools were tested using four different datasets with varying read coverages, read lengths and indel size ranges and that were produced with either the Illumina HiSeq 2000, HiSeq 2500, MiSeq v3, HiSeq PE-101 or NextSeq 500 v2 sequencing platforms. The semi-simulated WGS dataset with three different coverages (5×, 30×, and 60×) and two different read lengths (100bp and 250bp) included small indels and large indels from 1bp to 5000bp. Since to our knowledge there is no single unbiased real world benchmarking dataset that would cover both small and large indels, we chose to use three real-world datasets that represented WGS, WES and targeted gene panel sequencing data that together, cover small and large indels.

Our results demonstrated that the indel calling results vary greatly between indels depending on the size of the indels and the properties of the sequencing data. Deep convolutional neural network and random forest model-based tools, DeepVariant and Strelka2, demonstrated superior performance with small indel calling. However, their optimum indel call size range was limited to < 50bp. Split-read and paired-end reads-based algorithms (DELLY and Pindel) could detect the breakpoints of deletions efficiently from the mapping information of sequencing reads. Although the precision rates and recall rates of large deletion callings were relatively lower than those of small deletion callings, these tools were best for deletion calling with various types of sequencing data. The *de novo* sequence assembly-based tool, FermiKit, had the best performance for large insertion calling. Due to the read length limitation of NGS, the *de novo* sequence assembly algorithm could still be the best algorithm for detecting large novel insertions in human genomes with WGS data even though the performance of large insertion calling was not as good as that of small insertion calling. The local re-assembly methods (GATK HC and Platypus) were good for genotype-level indel calling. With the re-assembling of reads around indels, the context of variants could be better discovered, thus achieving greater genotype-level accuracies. Based on the F1 scores (S7 Table), the recommended variant calling tools for different sizes of indels, and different sequencing types are shown in Table 4.

**Table 4:** Recommended variant calling tools for different sized indels and sequencing types.

Data	Small indels (< 50bp)	Large indels (≥ 50bp)
WGS (5×)	DeepVariant, Platypus, Strelka2	DELLY, Pindel (only deletions)
WGS (30×,60×)	GATK HC, Platypus, Strelka2	DELLY, FermiKit, Pindel
WES (135×)	DeepVariant, GATK HC, Strelka2	Not evaluated

We evaluated the indel calling abilities and running times of our candidate tools. In addition, we also dissected the source of the FP indel calls. From our results, it can be seen that the majority of the FP calls came from SR regions, which may be due to the ambiguous sequence patterns around SR regions. Putative problems include the misalignment and inconsistent representation of variants in mapping and variant calling steps, respectively. Nonetheless, we still may have underestimated the influence of SR regions because the difficulties of sequencing SR regions for sequencing platforms are not easily reproduced in our semi-simulated WGS dataset. In real sequencing data, even though the difficulties of sequencing error-prone regions can be reproduced, it is still difficult for different callers to reach a consensus with short reads, thus these regions are generally excluded from the confidence regions of high-confidence variant calls, such as [55]⁠. Furthermore, in our semi-simulated WGS dataset, we shifted all the indels in the truth set to the left-most positions before the indel calling processes because we considered any post-normalization steps with tool-detected indels such as left-aligning to be an extra burden for users; moreover, some tools may gain an extra advantage and thus make the evaluation unfair for the other tools. Even though we tried to avoid any inconsistent representation of indels by left-aligning all the indels in our truth set, it still might be that we underestimated the complexity of the human genome and further efforts would be required to investigate the ambiguity of indels in human genome. Our semi-simulated genome represented the actual distribution of the indels in the human genome with the fact that the number of large indels is smaller than that of small indels. Further efforts would be required to create comprehensive datasets for large indel calling benchmarking.

In our evaluation, the majority of the tools were implemented with default parameters, and only minor changes were introduced when necessary. It is possible that the tools would benefit from optimized parameters to obtain more accurate results [56], but default parameters are the general starting point for tool usage and allow an equivalent assessment of the performance of the tools. Moreover, optimization requires the modification of all possible combinations of the parameters, which is time-consuming and impractical [57]. Datasets may require different combinations of parameters, which makes it even more of a challenge to attain the universal best combination. Experienced users may have prior knowledge of how to optimize the tools, based on their data properties to obtain the best performance. In this study, we considered that a user would use a given tool with its default parameters.

In general, higher coverage led to better results. The recall rates improved from 5× to 30×, then to 60×, especially for large indels. Fang et al. [58] suggested that 60× is the suitable sequencing coverage for WGS data from the HiSeq platform, however, in our study, we discovered that with the improved algorithms of indel calling methods in recent years, 30× sequencing coverage is also suitable in indel calling based on the performance of the tools. Our study showed no large differences in indel calling between 30× and 60× sequencing coverage data. Longer read length sequencing data was shown to help to call larger indels more accurately. In addition, longer read lengths may also contribute to alignment quality enabling more precise indel breakpoint detections. In our evaluation it was shown that indel calling for small size indels (< 50bp) was improved when machine learning-based methods were applied. Although the precision and recall were still not quite on the same level as for the SNVs, the indel calling concerning small indels (< 50bp) was assuring. For large indels, *de novo* assembly method was the best for calling large insertions, and split-read methods and paired-end reads methods were best for calling large deletions. In general, the genotyping abilities of tools for small indels were better than those for large indels. Future methodological development may benefit from improved machine learning models trained for large indel calling and should also integrate other methods such as *de novo* assembly methods to call both small and large indels with precise positions, sizes, and genotypes. The potential influence and features of library preparation, sample handling, and sequencing platform should also be considered in tool development. There is also room for further indel calling tool development for detection of large somatic indels when sequencing data is only available from a tumor sample.

## Supporting information

S1 Fig An example of ambiguous variant. A deletion of “CA” was mutated in a simple repeat region with repeated pattern “CA”. In the truth set, the deletion was represented with left-align manner, but in tool prediction, the deletion was represented with right-align manner. These inconsistent representations caused a same single variant reported with different positions, reference alleles and alternative alleles, further causing trouble for evaluation.
(TIF)

S2 Fig Distribution of 10bp deletions by their positional ambiguities. The red bars represent HuRef genome deletions and the blue bars represent deletions that were inserted at random sites.
(TIF)

S3 Fig Indel size distribution of Genome in a Bottle NA24385 WES dataset. (TIFF)

S4 Fig Indel size distribution of CHM1 cell line WGS dataset between 50bp – 10000bp. The proportion of deletions and insertions are marked as blue and green, respectively.
(TIFF)

S5 Fig Precision rates, recall rates and F1 scores of the tools for indels 30bp – 70bp using the semi-simulated datasets. (A) 5× coverage, 100bp read length sequencing data. (B) 30× coverage, 100bp read length sequencing data. (C) 60× coverage, 100bp read length sequencing data. (D) 30× coverage, 250bp read length sequencing data.
(TIF)

S6 Fig Precision rates, recall rates and F1 scores of the tools evaluated without genotype-match for deletions using the semi-simulated datasets. (A) 5× coverage, 100bp read length sequencing data. (B) 30× coverage, 100bp read length sequencing data. (C) 60× coverage, 100bp read length sequencing data. (D) 30× coverage, 250bp read length sequencing data.
(TIF)

S7 Fig Precision rates, recall rates and F1 scores of the tools evaluated without genotype-match for insertions using the semi-simulated datasets. (A) 5× coverage, 100bp read length sequencing data. (B) 30× coverage, 100bp read length sequencing data. (C) 60× coverage, 100bp read length sequencing data. (D) 30× coverage, 250bp read length sequencing data.
(TIF)

S8 Fig Precision rates, recall rates and F1 scores of the tools evaluated without genotype-match for deletions < 50bp using the semi-simulated datasets. (A) 5× coverage, 100bp read length sequencing data. (B) 30× coverage, 100bp read length sequencing data. (C) 60× coverage, 100bp read length sequencing data. (D) 30× coverage, 250bp read length sequencing data.
(TIF)

S9 Fig Precision rates, recall rates and F1 scores of the tools evaluated without genotype-match for insertions < 50bp using the semi-simulated datasets. (A) 5× coverage, 100bp read length sequencing data. (B) 30× coverage, 100bp read length sequencing data. (C) 60× coverage, 100bp read length sequencing data. (D) 30× coverage, 250bp read length sequencing data.
(TIF)

S10 Fig Homozygous and heterozygous precisions of variant calling tools using the semi-simulated datasets for small indels (< 50bp). (A) 5× coverage, 100bp read length sequencing data. (B) 30× coverage, 100bp read length sequencing data. (C) 60× coverage, 100bp read length sequencing data. (D) 30× coverage, 250bp read length sequencing data.
(TIF)

S11 Fig Homozygous precisions and heterozygous of precisions variant calling tools using the semi-simulated datasets for large indels (≥ 50bp). (A) 5× coverage, 100bp read length sequencing data. (B) 30× coverage, 100bp read length sequencing data. (C) 60× coverage, 100bp read length sequencing data. (D) 30× coverage, 250bp read length sequencing data.
(TIF)

S12 Fig Example of false genotyping caused by ambiguous regions. A homozygous deletion “chr1:878906 CTTT → C” was overlapped with 22 repeated “T” from chr1:878907-878928. The read that fully covered the repeat region had a 3bp gap in the CIGAR section of the BAM file. The read in which only the head or tail overlapped with the repeat region preferred to shorten its head or tail to omit the gap, based on the alignment algorithm. Simply counting the numbers of alleles at this site may lead to a low allele frequency, which then causes the tool to make a mistake and call a homozygous deletion a heterozygous one.
(TIF)

S1 Table Indel size distribution of the semi-simulated WGS dataset used for tool evaluation. Deletions and insertions are shown separately with different size ranges.
(XLSX)

S2 Table The characteristics of indels in targeted gene panel sequencing dataset. (XLSX)

S3 Table The TPs, FPs, FNs, precision rates, recall rates and F1 scores of variant calling tool for all size ranges of deletions and insertions with the semi-simulated sequencing datasets. (XLSX)

S4 Table Multiallelic tool-detected indels and non-valid genotype indel calls of variant calling results with semi-simulated sequencing datasets. The values are total numbers of tool-detected indels of each type. The values in brackets are numbers of indels which passed position-match and size-match but failed with genotype-match.
(XLSX)

S5 Table Evaluation results of variant calling tools with GIAB NA24385 WES data. Summary evaluation results of variant calling tools with GIAB NA24358 WES data. Evaluation results were generated by hap.py.
(XLSX)

S6 Table The TPs, FPs, FNs, FDRs, and sensitivities of variant calling tools with CHM1 cell line WGS data. (XLSX)

S7 Table The TPs, FPs, FNs, precision rates, recall rates and F1 scores of variant calling tools for small and large indels with the semi-simulated sequencing datasets. (XLSX)

S1 File Supplementary methods descriptions. (DOCX)
