# Supplementary figures and images for: Tool evaluation for the detection of variably sized indels from next generation whole genome and targeted sequencing data
Source: PLoS Comput Biol. 2022 Feb 17;18(2):e1009269. doi: 10.1371/journal.pcbi.1009269 (PMC8916674; doi:10.1371/journal.pcbi.1009269)

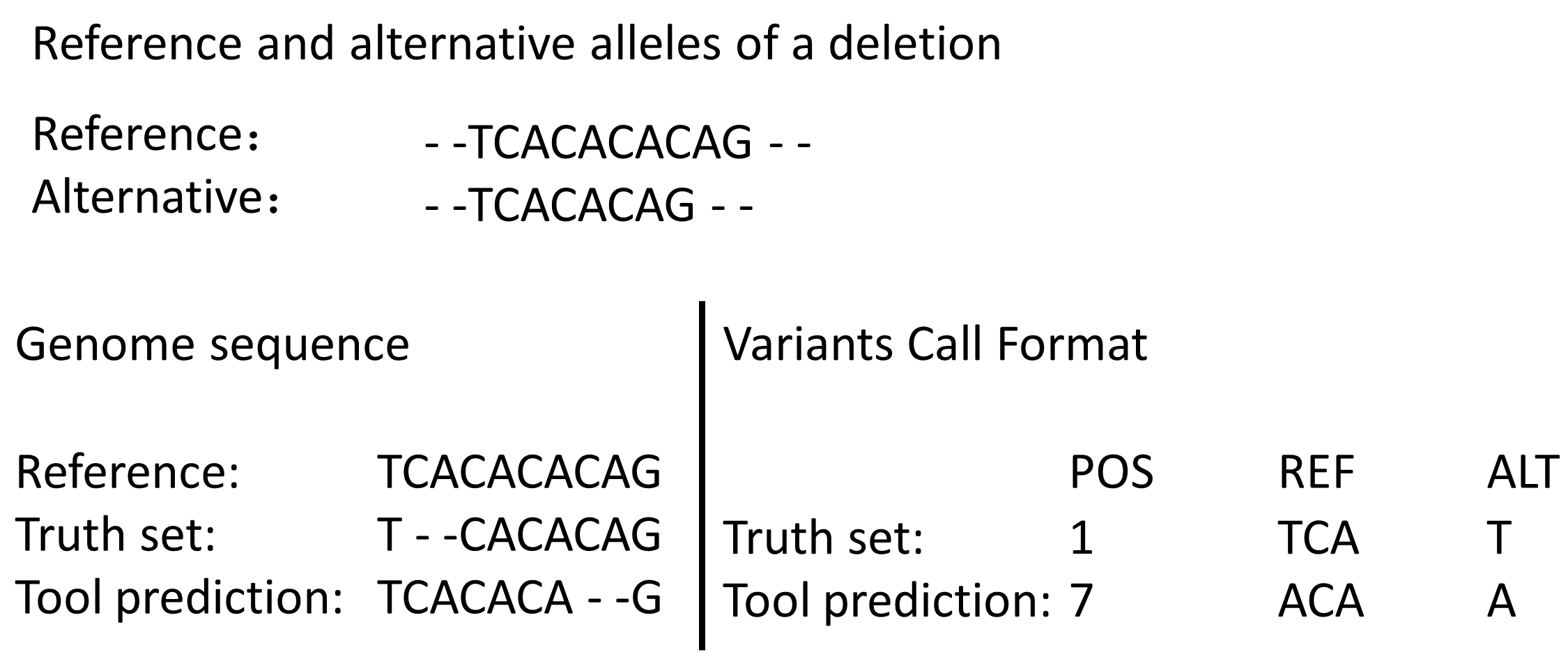

Supplement: S1 Fig — A deletion of “CA” was mutated in a simple repeat region with repeated pattern “CA”. In the truth set, the deletion was represented with left-align manner, but in tool prediction, the deletion was represented with right-align manner. These inconsistent representations caused a same single variant reported with different positions, reference alleles and alternative alleles, further causing trouble for evaluation. (TIF) [file pcbi.1009269.s001.tif]

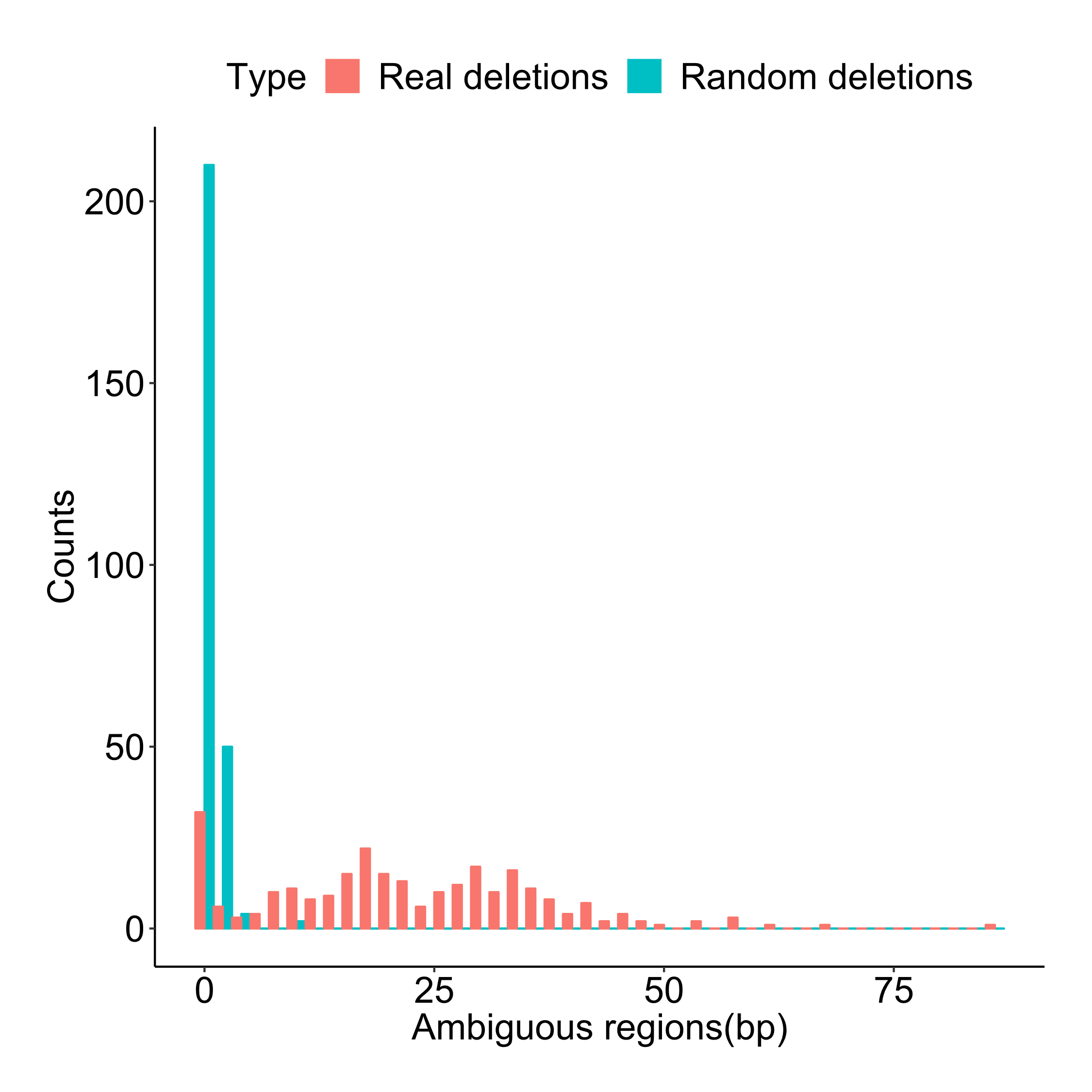

Supplement: S2 Fig — The red bars represent HuRef genome deletions and the blue bars represent deletions that were inserted at random sites. (TIF) [file pcbi.1009269.s002.tif]

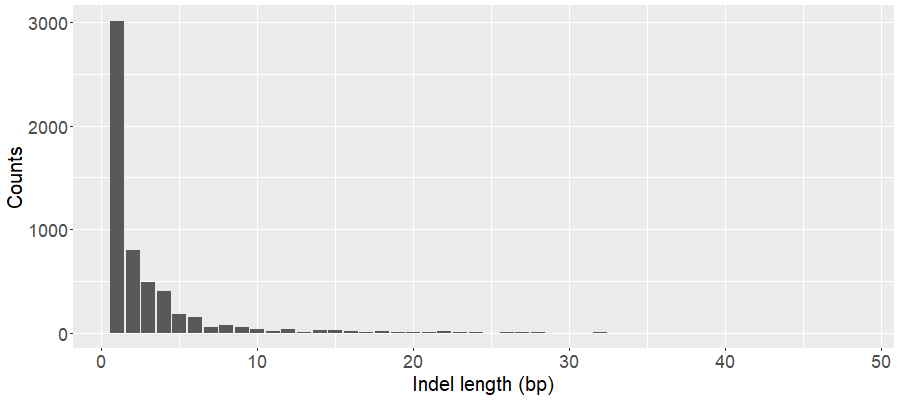

Supplement: S3 Fig — (TIFF) [file pcbi.1009269.s003.tiff]

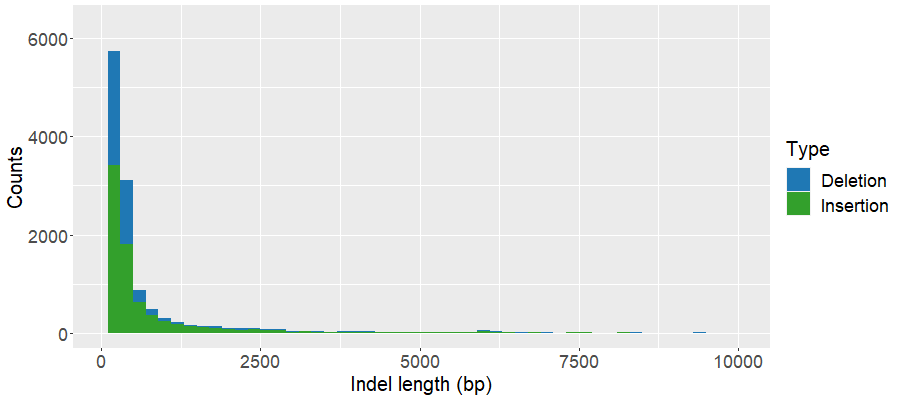

Supplement: S4 Fig — The proportion of deletions and insertions are marked as blue and green, respectively. (TIFF) [file pcbi.1009269.s004.tiff]

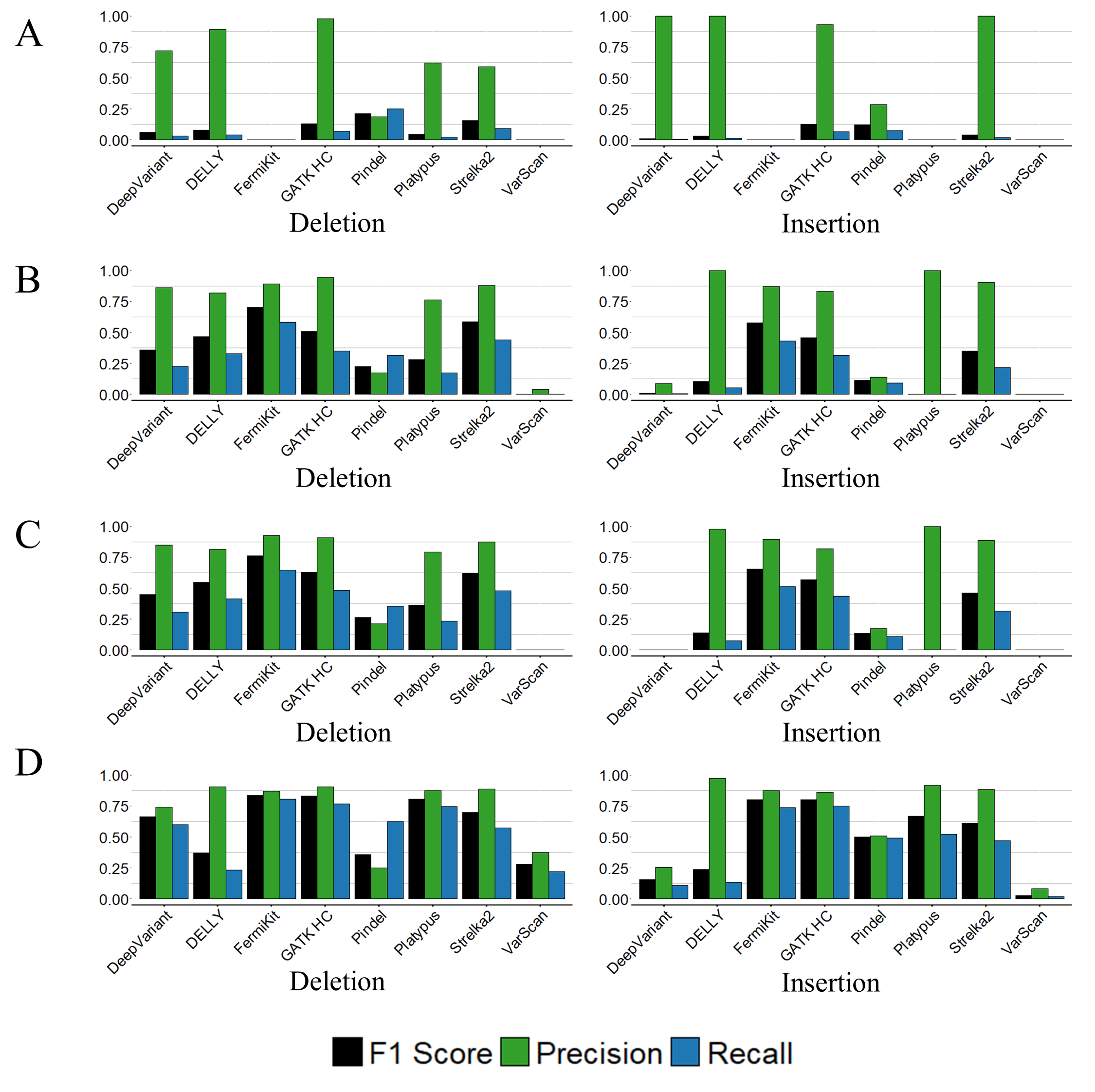

Supplement: S5 Fig — (A) 5× coverage, 100bp read length sequencing data. (B) 30× coverage, 100bp read length sequencing data. (C) 60× coverage, 100bp read length sequencing data. (D) 30× coverage, 250bp read length sequencing data. (TIF) [file pcbi.1009269.s005.tif]

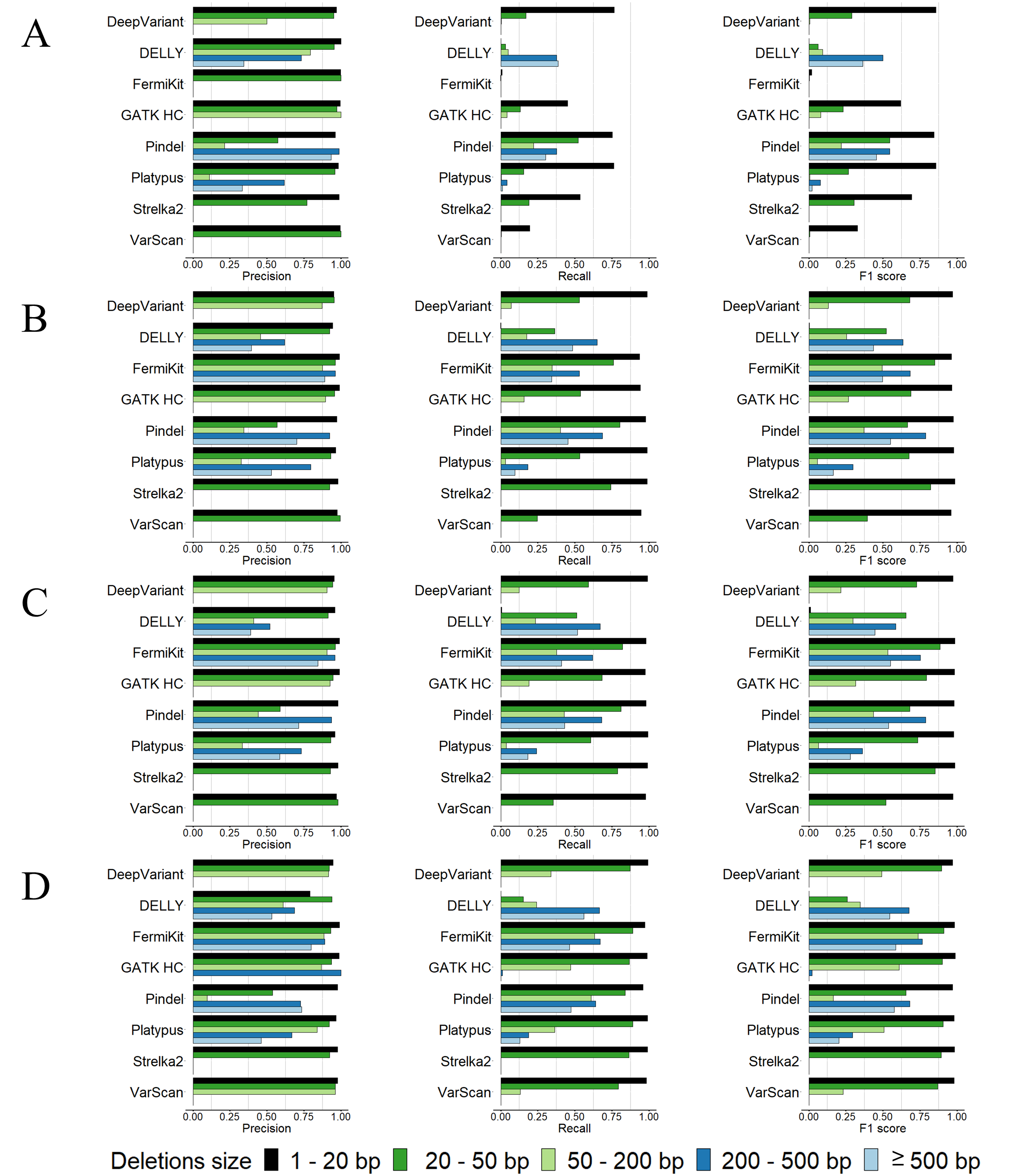

Supplement: S6 Fig — (A) 5× coverage, 100bp read length sequencing data. (B) 30× coverage, 100bp read length sequencing data. (C) 60× coverage, 100bp read length sequencing data. (D) 30× coverage, 250bp read length sequencing data. (TIF) [file pcbi.1009269.s006.TIF]

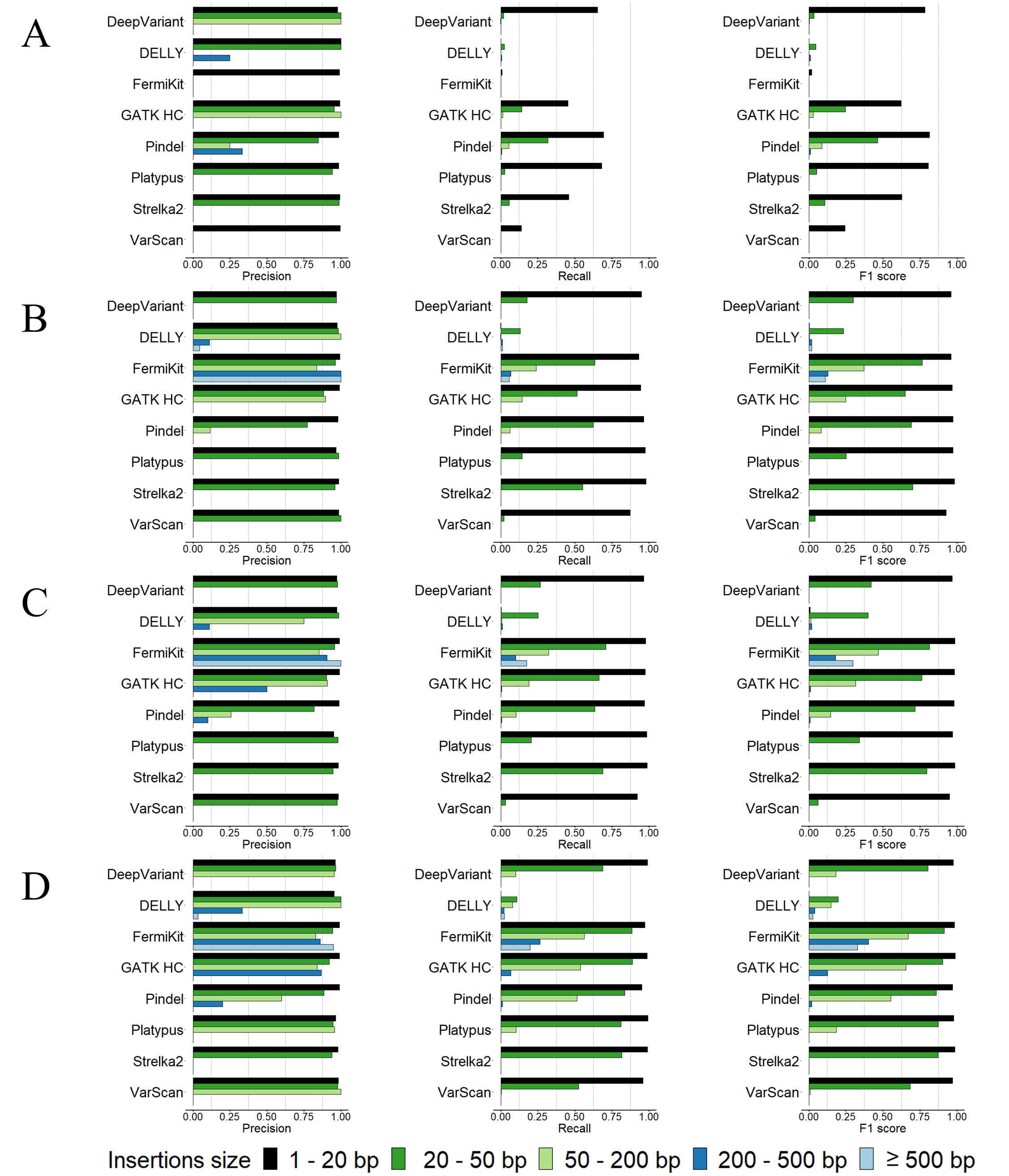

Supplement: S7 Fig — (A) 5× coverage, 100bp read length sequencing data. (B) 30× coverage, 100bp read length sequencing data. (C) 60× coverage, 100bp read length sequencing data. (D) 30× coverage, 250bp read length sequencing data. (TIF) [file pcbi.1009269.s007.TIF]

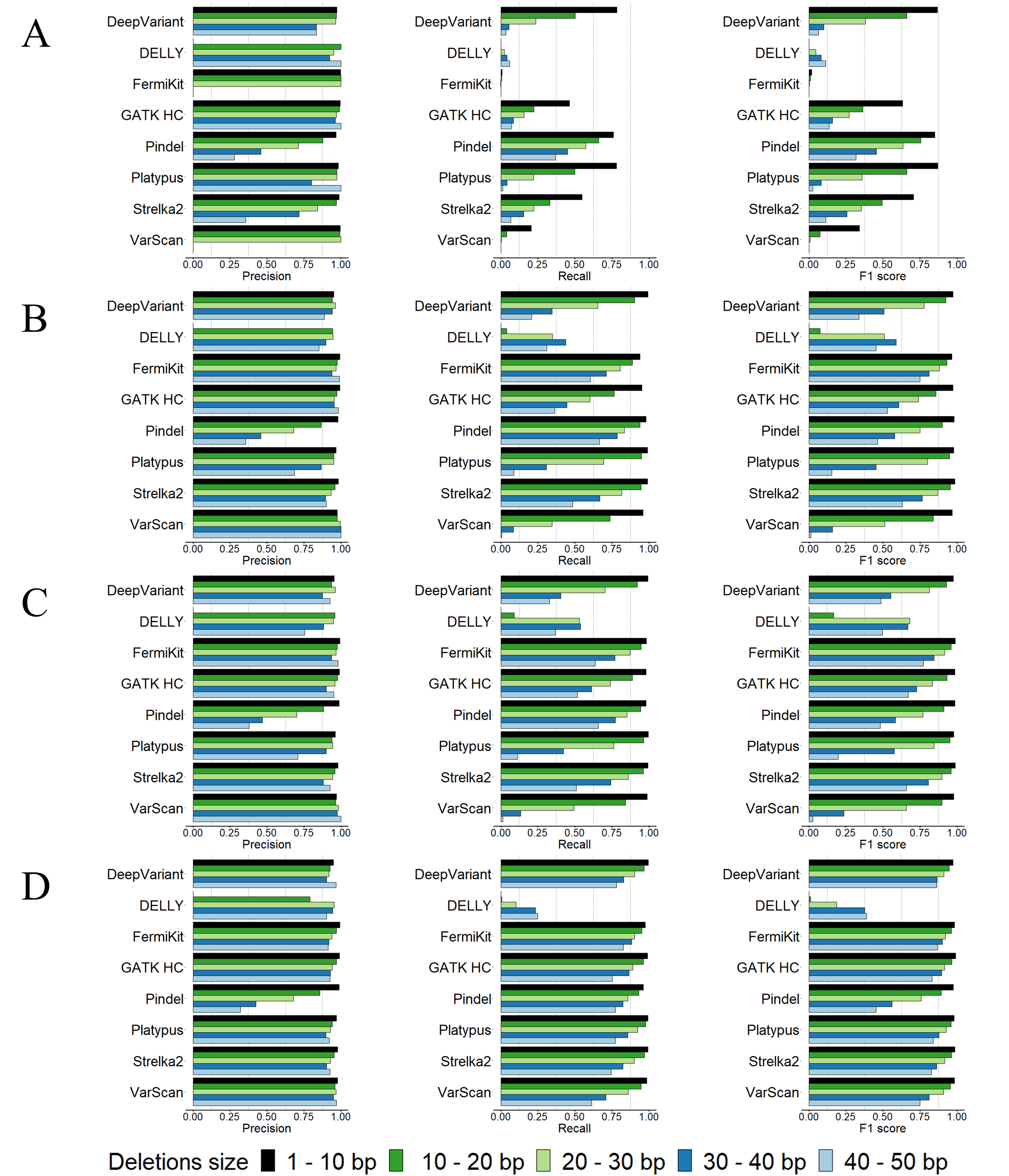

Supplement: S8 Fig — (A) 5× coverage, 100bp read length sequencing data. (B) 30× coverage, 100bp read length sequencing data. (C) 60× coverage, 100bp read length sequencing data. (D) 30× coverage, 250bp read length sequencing data. (TIF) [file pcbi.1009269.s008.TIF]

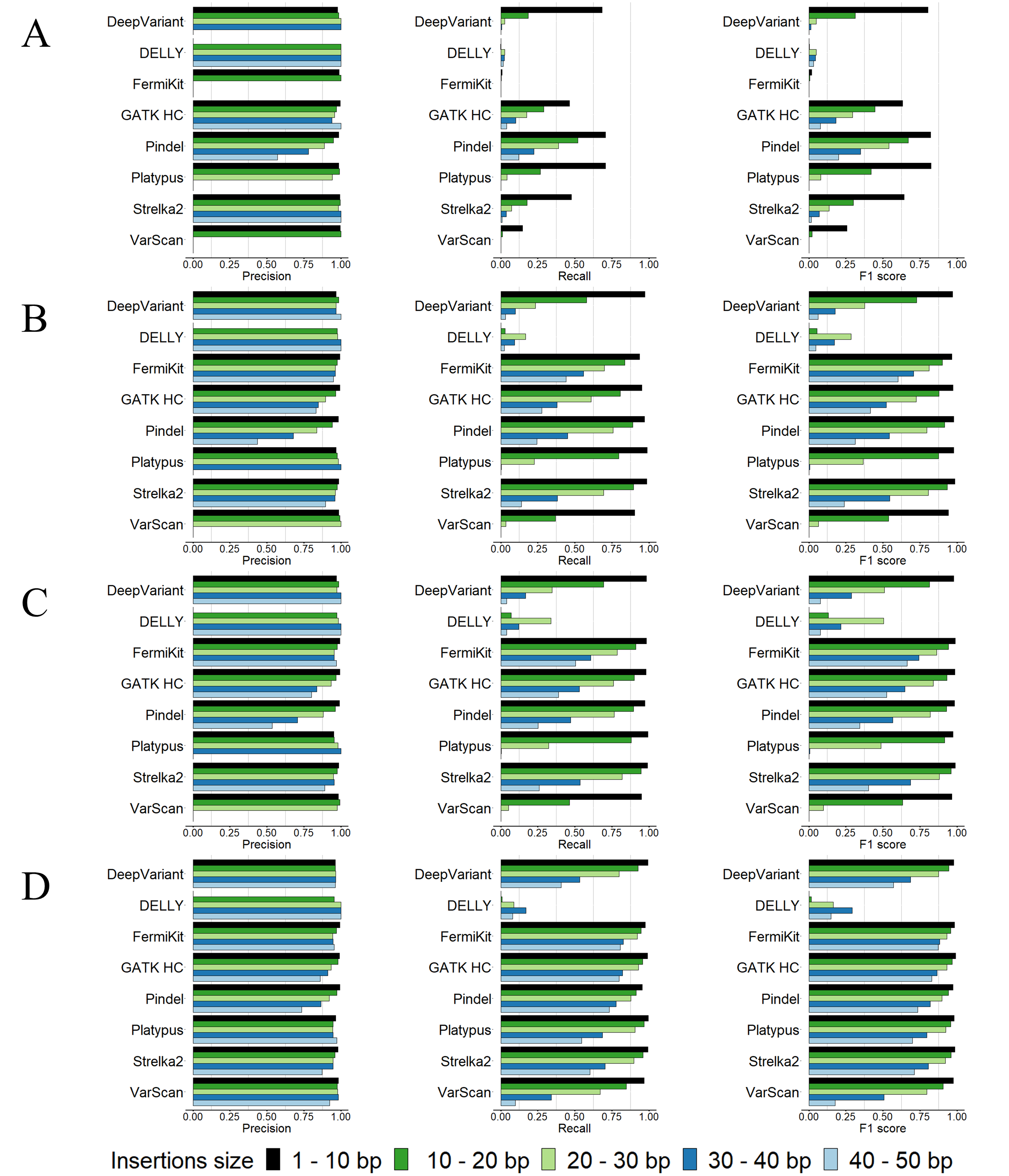

Supplement: S9 Fig — (A) 5× coverage, 100bp read length sequencing data. (B) 30× coverage, 100bp read length sequencing data. (C) 60× coverage, 100bp read length sequencing data. (D) 30× coverage, 250bp read length sequencing data. (TIF) [file pcbi.1009269.s009.TIF]

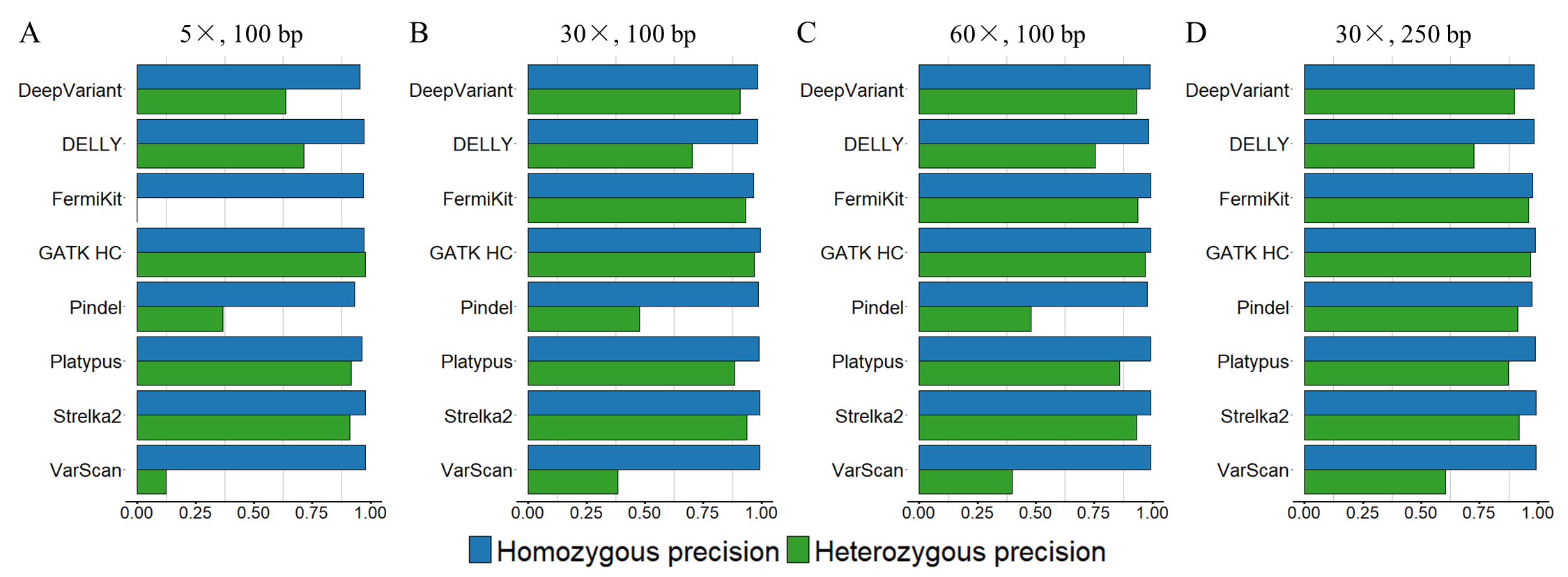

Supplement: S10 Fig — (A) 5× coverage, 100bp read length sequencing data. (B) 30× coverage, 100bp read length sequencing data. (C) 60× coverage, 100bp read length sequencing data. (D) 30× coverage, 250bp read length sequencing data. (TIF) [file pcbi.1009269.s010.TIF]

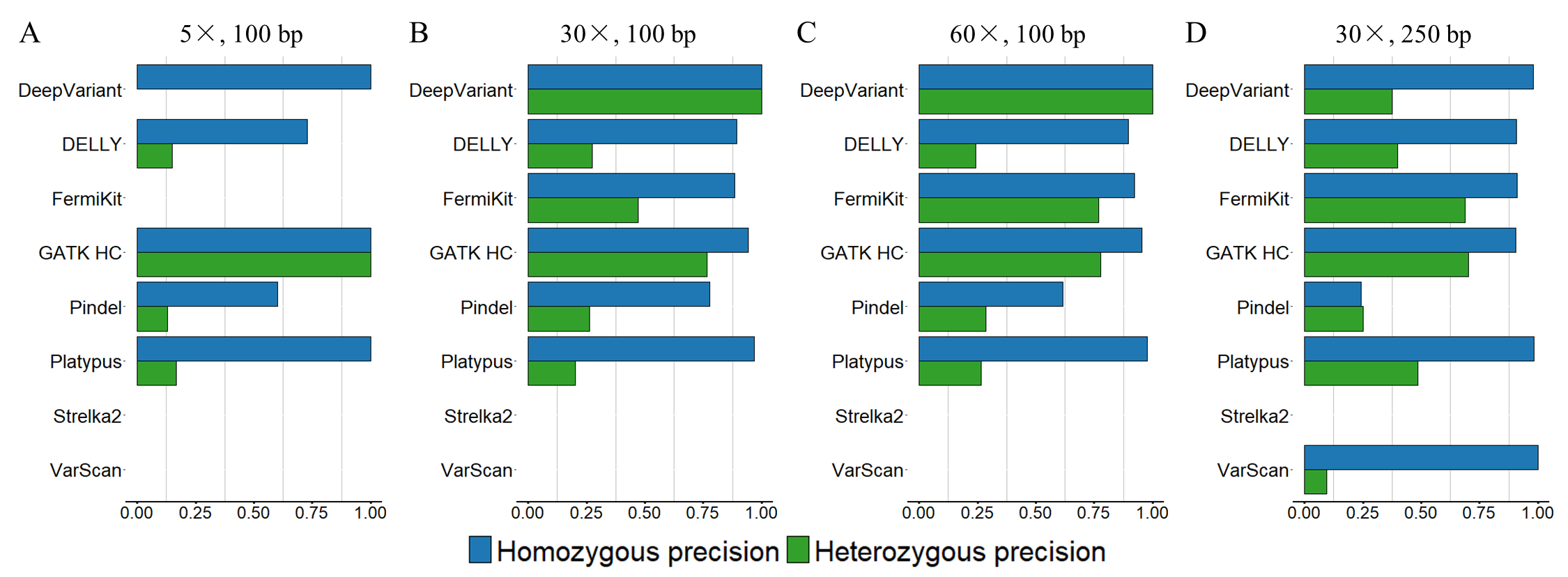

Supplement: S11 Fig — (A) 5× coverage, 100bp read length sequencing data. (B) 30× coverage, 100bp read length sequencing data. (C) 60× coverage, 100bp read length sequencing data. (D) 30× coverage, 250bp read length sequencing data. (TIF) [file pcbi.1009269.s011.TIF]

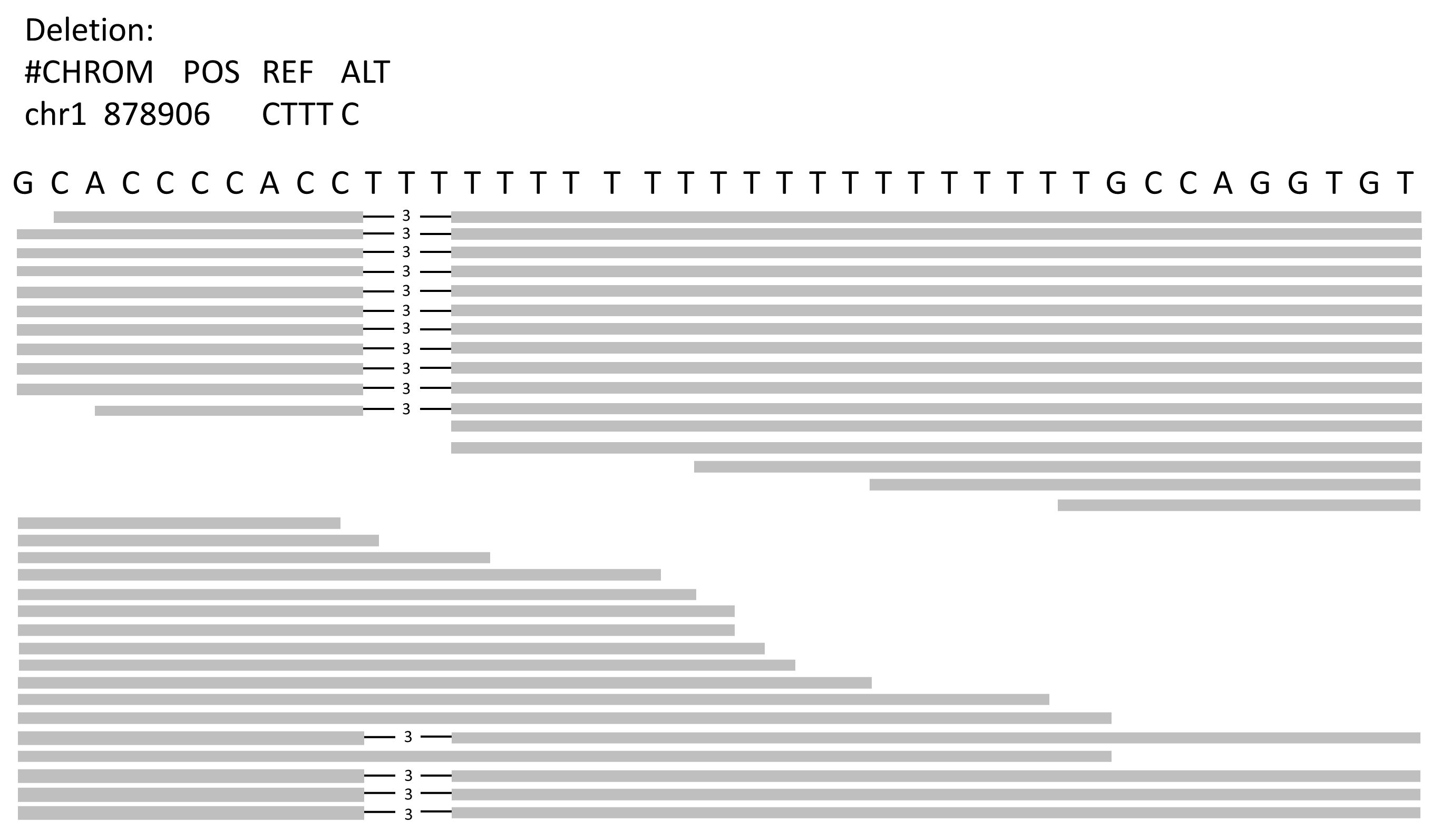

Supplement: S12 Fig — A homozygous deletion “chr1:878906 CTTT → C” was overlapped with 22 repeated “T” from chr1:878907-878928. The read that fully covered the repeat region had a 3bp gap in the CIGAR section of the BAM file. The read in which only the head or tail overlapped with the repeat region preferred to shorten its head or tail to omit the gap, based on the alignment algorithm. Simply counting the numbers of alleles at this site may lead to a low allele frequency, which then causes the tool to make a mistake and call a homozygous deletion a heterozygous one. (TIF) [file pcbi.1009269.s012.tif]
