# Supplementary material for: Tool evaluation for the detection of variably sized indels from next generation whole genome and targeted sequencing data
Source: PLoS Comput Biol. 2022 Feb 17;18(2):e1009269. doi: 10.1371/journal.pcbi.1009269 (PMC8916674; doi:10.1371/journal.pcbi.1009269)
Supplement: S1 File — (DOCX) [file pcbi.1009269.s020.docx]

**Tool evaluation for the detection of variably sized indels from next generation whole genome and targeted sequencing data**

Ning Wang^1^, Vladislav Lysenkov^1^, Katri Orte^2,3^, Veli Kairisto^3^, Juhani Aakko^1^, Sofia Khan^1*^, Laura L. Elo^1,4*^

^1^Turku Bioscience Centre, University of Turku and Åbo Akademi University, Turku, Finland.

^2^Department of Pathology, Laboratory Division, Turku University Hospital, Turku, Finland

^3^Department of Genomics, Laboratory Division, Turku University Hospital, Turku, Finland

^4^Institute of Biomedicine, University of Turku, Finland

*To whom correspondence should be addressed.

Corresponding author:

* laura.elo@utu.fi (LLE); * sofia.khan@utu.fi (SK)

# Supplementary methods

[Method of building semi-simulated WGS data 1](#_Toc75264365)

[Tool implementation 3](#_Toc75264366)

[Data pre-processing pipeline 3](#_Toc75264367)

[Methods of evaluation 4](#_Toc75264368)

[Supplementary references 5](#_Toc75264369)

### Method of building semi-simulated WGS data

We reconstructed chromosome 1 and 2 of the HuRef genome, based on hg19, by similar methods to [1,2], which we describe here in detail. First, we downloaded the variants file of HuRef and only kept indels from chromosome 1 and 2. Then, we extracted the start and end positions of each indel and used the UCSC liftOver tool to convert HuRef indels from hg18 to hg19. To avoid complicated evaluation criteria caused by ambiguous variants which are actually the same variants but with inconsistent representations in tool predictions and truth set, we normalised the HuRef variants using a similar algorithm as in the software tool, vt [3]. After normalization, we filtered out complex variants whose ambiguous regions were overlapping and may have caused inconsistent genotypes. Due to the small number of indels > 5000bp, which may limit the accuracy of evaluation results, we only used indels < 5000bp in our evaluation study. In total, 43,066 insertions and 45,223 deletions, ranging from 1bp up to 5000bp were included in our evaluation study. The codes of generating semi-simulated genome were available at <https://github.com/elolab/semi-simulated_indel_dataset>.

In our truth set, around 30% of the indels (26,301 indels) were located in simple repeat regions, annotated by the UCSC genome browser. Additionally, after variant normalization processes, we found that around 75% of the indels (66,146 indels) were ambiguous variants which has at least one duplicated indel pattern located in the original (HuRef) positions. This indicates that the majority of the indels were located in a sequence context where similar patterns were adjacent. To compare our semi-simulated genome with a fully simulated genome where indels were randomly inserted, we chose 10bp deletions from the chromosome 1 of our semi-simulated genome and created the same number of randomly inserted 10bp deletions, and then compared the sizes of ambiguous regions around the variants. The proportion of ambiguous deletions from our semi-simulated genome were higher than the deletions from the fully simulated genome with indels randomly inserted (S2 Fig). This indicated that indels from our semi-simulated genome were better able to reflect the complexity of the human genome than randomly inserted indels.

We used chromosome 1 and 2 of hg19 as our reference genome, and a modified version of svsim [4] (https://github.com/mfranberg/svsim) to insert each indel. In order to keep the ambiguity of indels, we kept the original sequences of insertions, instead of the random sequences. We created a simulated genome with two different haplotypes. First, we randomly chose two subsets of variants from different size ranges and only inserted them into one of the haplotypes (S1 Table). Then the remaining variants were inserted into both of the haplotypes. These variants were treated as heterozygous and homozygous variants, respectively. The detailed commands of building the semi-simulated WGS sequencing data are available at <https://github.com/elolab/semi-simulated_indel_dataset>.

### Tool implementation

We evaluated indel calling tools using default parameters, with some necessary adjustments of parameters that were needed to make the tools run properly. DELLY was activated as small indel calling (-i flag) for all semi-simulated WGS datasets. For Pindel, as the default minimum support reads to report an indel was set to one, Pindel is very aggressive to detect potential indels and produce too many false positive results. Even in the original publication of Pindel, the authors did not use the default parameters [5]. A previous study [6] optimized the parameters of Pindel. Accordingly, we calculated the ratio between minimum support reads and overall coverage based on the results of [6] , and applied the recommended minimum support reads to two real sequencing datasets and three semi-simulated WGS sequencing datasets (30× 100bp, 30× 250bp and 60× 100bp). For 5× coverage sequencing data, we used a value of two for Pindel minimum support reads to report an indel and provide genotype (-M and -mc flag), according to the original publication [5]. The maximum variant size of Platypus was set to 8000 for all semi-simulated WGS datasets and 20000 for CHM1 cell line WGS data. Platypus has a built-in module called “Cortex” that does local haplotype assembly for variant calling, which we activated by a command line setting (assemble=1) for all semi-simulated datasets and CHM1 cell line WGS data but de-activated for GIAB NA24385 WES data and targeted gene panel sequencing data. The minimum coverage for reporting a variant in VarScan was set to two for 5× semi-simulated WGS data. Detailed tool running parameters are available at <https://github.com/elolab/semi-simulated_indel_dataset>.

### Data pre-processing pipeline

We used a fixed bioinformatics pipeline to pre-process both semi-simulated and real sequencing reads. FASTQC (https://www.bioinformatics.babraham.ac.uk/projects/fastqc/) and Trim Galore! (https://www.bioinformatics.babraham.ac.uk/projects/trim_galore/) were used for quality control and refinement. We kept reads >20bp and a quality score of 25. The alignment of all the sequencing data was performed by BWA-MEM [7], followed by sorting and indexing by SAMtools [8]. The semi-simulated WGS data was aligned with chromosome 1 and 2 of the human reference genome hg19, the CHM1 data was aligned against hg19, and the NA24385 whole exome data was aligned against hs37d5. Minor modifications were applied to alignment files, such as the mpileup process for VarScan by SAMtools, and adding artificial read group information for GATK HC and Platypus using Picard tools [9]. As the semi-simulated WGS data was not generated from a real sequencing platform we were not able to fully follow the guidelines of GATK best practices. Therefore, base quality score recalibration (BQSR) and variant quality score recalibration (VQSR) were not applied when processing semi-simulated WGS data. The execution of the fixed bioinformatics pipeline for all the datasets is available at <https://github.com/elolab/semi-simulated_indel_dataset>.

### Methods of evaluation

The format of all the outputs of tools was VCF format.

For the evaluation of tools with the semi-simulated data, only positions and sizes of tool-detected indels were needed. Different extraction methods were applied for different tools. For DeepVariant, GATK HC, Platypus, Strelka2 add VarScan, the positions of tool-detected indels were directly extracted from the “POS” column of VCF format outputs, the sizes of tool-detected indels were calculated with the numbers of nucleotides from “REF” and “ALT” columns. For DELLY, the “SVTYPE” from INFO column was used to extract indel types. “DEL” was used as deletion, “INS” and “DUP” were used as insertion in our study. For “DEL” and “DUP”, “END” information from INFO column was used together with the position of tool-detected indel to calculate indel size. For “INS”, “INSLEN” information from INFO column was used as indel size. For FermiKit, two VCF files for small indels and large indels were reported as outputs. For the small indel VCF file, the positions of tool-detected indels were extracted from the “POS” column of the small indels VCF format output, the sizes of tool-detected indels were calculated based on the numbers of characters of the “REF” and the “ALT” columns for deletions and insertions, respectively. For the large indels VCF format output, “SVTYPE” from INFO column was used for extracting deletion or insertion, “SVLEN” from INFO column was used as the indel size. For Pindel, the positions of tool-detected indels were extracted from the VCF output and indel sizes were extracted from “SVLEN” in INFO column. Due to Pindel long insertions VCF output file “LI.vcf” contained no insertions size information, so this file was not considered in our evaluation.

We used custom R scripts to evaluate each tool output of semi-simulated sequencing data with truth set by applying position-match, size-match and genotype-match as our criteria. The custom R scripts are available at <https://github.com/elolab/semi-simulated_indel_dataset>.

For evaluation of tools with GIAB NA24385 WES data, the VCF outputs of tools were directly used as inputs for hap.py. For FermiKit, only the small indel VCF file was used due to large indel VCF file not containing full information for using hap.py. For Pindel, the deletions, insertions and duplications from their corresponding VCF outputs were combined together as the input file for hap.py, after filtering indels larger than 50bp.

The command line for hap.py (v0.3.10-2-gfd67904) was:

/hap.py-build/bin/hap.py HG002_GRCh37_GIAB_highconf_CG-IllFB-IllGATKHC-Ion-10X-SO

LID_CHROM1-22_v.3.3.2_highconf_triophased.vcf tool.vcf -f HG002_GRCh37_GIAB_highcon

f_CG-IllFB-IllGATKHC-Ion-10X-SOLID_CHROM1-22_v.3.3.2_highconf_noinconsistent.bed -T exome-target-region.bed.gz -V -X -L --gender male -o tool_evaluation -r Homo sapiens.GRCh37.

dna.primary_assembly.fa

For evaluation of tools with CHM1 cell line WGS data, only positions and sizes of tool-detected indels were needed. The extraction methods of tool-detected indels’ positions and sizes were the same as with the semi-simulated sequencing data. Since Pindel long insertions VCF output file “LI.vcf” contained no insertions size information, it was not considered in our evaluation. We used custom R and bash scripts to evaluate each tool output against the truth set. Only indels in chr1-chr22 and chrX with sizes ≥50bp and < 10000bp were considered. In our criteria, we allowed that one tool-detected indel matched several the truth set indels or one truth set indel matched several tool-detected indels, which indicated the tool may split a truth indel into several small indels or merge several truth indels as a joint indel. We applied this loose criteria because suggested by previous research [1,10], the concordance between tool-detected indels from CHM1 cell line Illumina WGS data and the truth indels of PacBio sequencing data were low, indicating that the indel calling methodology for truth indels from CHM1 cell line WGS datasets has its own bias. The custom R and bash scripts are available at <https://github.com/elolab/semi-simulated_indel_dataset>.

### Supplementary references

1. Shrestha AMS, Frith MC, Asai K, Richard H. Jointly aligning a group of DNA reads improves accuracy of identifying large deletions. Nucleic Acids Res. 2018;46. doi:10.1093/nar/gkx1175

2. Talwalkar A, Liptrap J, Newcomb J, Hartl C, Terhorst J, Curtis K, et al. SMaSH: A benchmarking toolkit for human genome variant calling. Bioinformatics. 2014. doi:10.1093/bioinformatics/btu345

3. Tan A, Abecasis GR, Kang HM. Unified representation of genetic variants. Bioinformatics. 2015. doi:10.1093/bioinformatics/btv112

4. Mfranberg. Simulation toolbox for structural variations. In: Github [Internet]. 2013. Available: https://github.com/mfranberg/svsim

5. Ye K, Schulz MH, Long Q, Apweiler R, Ning Z. Pindel: A pattern growth approach to detect break points of large deletions and medium sized insertions from paired-end short reads. Bioinformatics. 2009;25: 2865–2871. doi:10.1093/bioinformatics/btp394

6. Ghoneim DH, Myers JR, Tuttle E, Paciorkowski AR. Comparison of insertion/deletion calling algorithms on human next-generation sequencing data. BMC Res Notes. 2014;7. doi:10.1186/1756-0500-7-864

7. Li H. Aligning sequence reads, clone sequences and assembly contigs with BWA-MEM. 2013;arXiv:1303. doi:arXiv:1303.3997 [q-bio.GN]

8. Li H, Handsaker B, Wysoker A, Fennell T, Ruan J, Homer N, et al. The Sequence Alignment/Map format and SAMtools. Bioinformatics. 2009;25: 2078–2079. doi:10.1093/bioinformatics/btp352

9. Broad Institute. Picard Toolkit. GitHub Repository. 2018. Available: http://broadinstitute.github.io/picard/

10. Kronenberg ZN, Osborne EJ, Cone KR, Kennedy BJ, Domyan ET, Shapiro MD, et al. Wham: Identifying Structural Variants of Biological Consequence. PLoS Comput Biol. 2015;11. doi:10.1371/journal.pcbi.1004572
